# Supplementary material for: Influence of the Topology of Poly(L-Cysteine) on the Self-Assembly, Encapsulation and Release Profile of Doxorubicin on Dual-Responsive Hybrid Polypeptides
Source: Pharmaceutics. 2023 Feb 27;15(3):790. doi: 10.3390/pharmaceutics15030790 (PMC10055909; doi:10.3390/pharmaceutics15030790)
Supplement: Supplementary file 1 [file pharmaceutics-15-00790-s001.zip › pharmaceutics-2202834-supplementary.pdf]

pharmaceutics

Supplementary Material: Influence of the Topology of Poly(L-Cysteine) on the Self-Assembly, Encapsulation and Release of Doxorubicin on Dual-Responsive Hybrid Polypeptides

Dimitra Stavroulaki, Iro Kyroglou, Dimitrios Skourtis, Varvara Athanasiou, Pandora Thimi, Sosanna Sofianopoulou, Diana Kazaryan, Panagiota G. Fragouli, Andromahi Labrianidou, Konstantinos Dimas, George Patias, David M. Haddleton and Hermis Iatrou

Synthesis of N-Carboxy Anhydrides

Synthesis of N^{im}-Trityl-L-Histidine N-Carboxy Anhydride

For the synthesis of N^{im}-*Trityl*-L-Histidine NCA a previously reported method of our group[1] was mainly followed with some modifications. The synthesis was conducted in two steps: In the first step the hydrochloric salt of N^{im}-*Trityl*-L-Histidine NCA was formed, and finally the desired pure N^{im}-*Trityl*-L-Histidine NCA was produced after the removal of the HCl.

A. Synthesis of N^{im}-Trityl-L-His-NCA·HCl salt

Firstly, 10.0 g of BOC-His(*Trt*)-OH (20.1 mmol, MB=497.58 g mol^{-1}) were placed in a 1000 mL two neck round-bottom flask and left for drying overnight in high vacuum line (HVL). Then 150 mL THF were distilled in the flask, giving a clear solution. The reaction flask was placed in an ice-bath, under continuous argon flow and the addition of 1.7 mL of thionyl chloride (23.43 mmol, MB=118.97 g mol^{-1}, d=1.64 g mL^{-1}) diluted in 30 mL THF was followed. The reaction lasted 2 hours and then the solution was precipitated in 2 L of $(Et)_2O$, giving the N^{im}-*Trityl*-L-His-NCA·HCl salt as the main product. The formed solid was separated by filtration through a borosilicate funnel Por 4, transferred to a 500 mL round bottom-flask and finally dried in HVL. The solid mixture was composed of N^{im}-*Trityl*-L-His-NCA·HCl salt, the pure anhydride and the initial substrate. In order to isolate the desired N^{im}-*Trityl*-L-His-NCA from the other components a recrystallization procedure was performed. Briefly, 250 mL ethyl acetate were distilled in the flask in HVL forming a suspension. Then the flask was removed from the HVL, filled with argon and placed into a water bath at 40 °C for 1 h, where the solution was cleared instantly for a few minutes and then turned turbid again. After that, the temperature was decreased at 0 °C, and the N^{im}-*Trityl*-L-His-NCA·HCl salt was precipitated, while the other components of the mixture remained diluted in ethyl acetate. Then a filtration procedure was conducted with the use of a Buchner system in order to isolate the desired N^{im}-*Trityl*-L-His-NCA·HCl salt. Finally, the NCA salt was transferred to a pre-weighted 100 mL round-bottom flask and dried overnight under high vacuum (5.238 g, 0.011 mol).

B. Synthesis of N^{im}-Trityl-L-His-NCA

The N^{im}-*Trityl*-L-His-NCA·HCl salt was weighed (5.238 g, 0.011 mol) and was transferred again to 500 mL two neck round-bottom flask, where 200 mL of EtOAc were distilled. The reaction flask was placed in an ice-bath at 0 °C, under continuous argon flow and the dropwise addition of a stoichiometric amount of distilled triethylamine (1.6 mL, 11.47 mmol, MB=101.19 g mol^{-1}, d=0.7255 g mL^{-1}) diluted in 30 mL of ethyl acetate was followed. After the completion of the addition, the reaction mixture was stirred for another 20 min. The resulting salt of triethylamine hydrochloride was separated from the solution mixture by filtration through a borosilicate funnel Por 4 and simultaneously the filtrate was poured in 2 L of distilled hexane in order to precipitate and recrystallize the N^{im}-*Trityl*-L-His-NCA. The desired white solid precipitate of N^{im}-*Trityl*-L-His-NCA was isolated by filtration through a Buchner system, dried overnight in HVL and stored in a

glove box (4.092 g, 9.7 mmol, 48%). The whole synthetic procedure was monitored by FT-IR spectroscopy, while the purity of *N^im^-Trityl*-L-His-NCA was confirmed by ^{1}H-NMR and FT-IR spectroscopy (**Figure S1, S2**). ^{1}H-NMR (600 MHz, $CDCl_3$, δ, ppm): 2.96–3.18 (c: 2H, -**C**H_2), 4.55–4.57 (b:1H, –C**H**– of the NCA ring), 6.66 (a: 1H, –N**H**– of the NCA ring), 7.08–7.35 (f: 16H, ArH of trityl group), 7.39 (d: 1H, -C=C**H**-N of the imidazole ring), 7.68 (e: 1H, -N=C**H**-N of the imidazole ring) (**Figure S1**). FT-IR (KBr, thin film): 1707 cm^{-1} (ν C=O, s precursor amino acid), 1855, 1781 cm^{-1} (ν C=O, s), 1619 cm^{-1}(ν NHCl salt, s), 752 cm^{-1}, 700 cm^{-1} (ν =C–H out of plane bend, s) (**Figure S2**). The reactions employed in the synthesis of *N^im^-Trityl*-L-His-NCA are shown in **Scheme S1**.

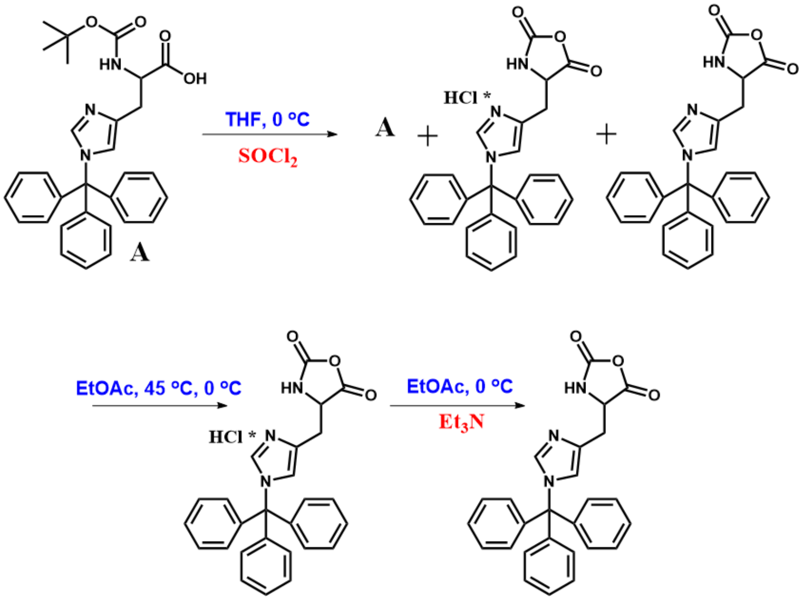

Scheme S1. The synthesis reaction of *N^im^-Trityl*-L-His-NCA.

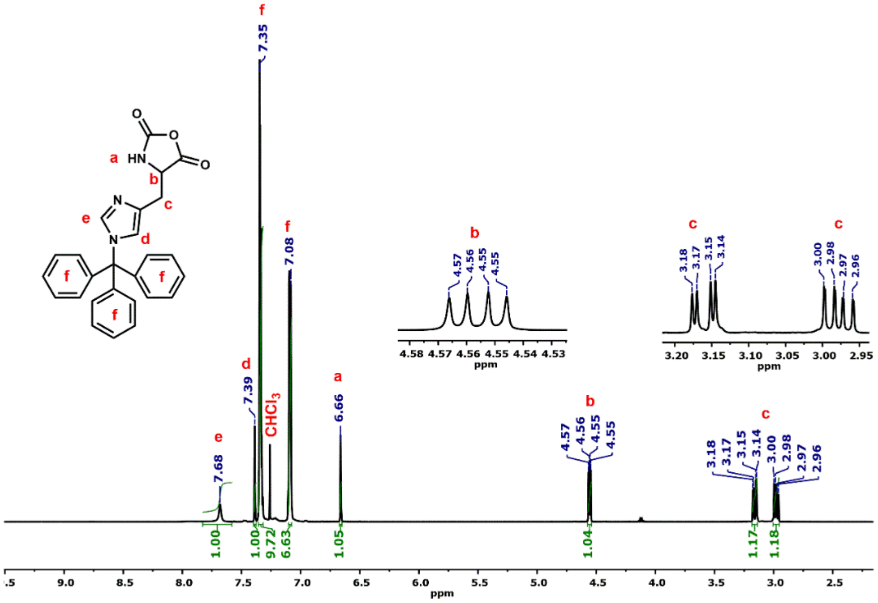

Figure S1. ^{1}H-NMR spectrum of the *N^im^-Trityl*-L-His-NCA in $CDCl_3$.

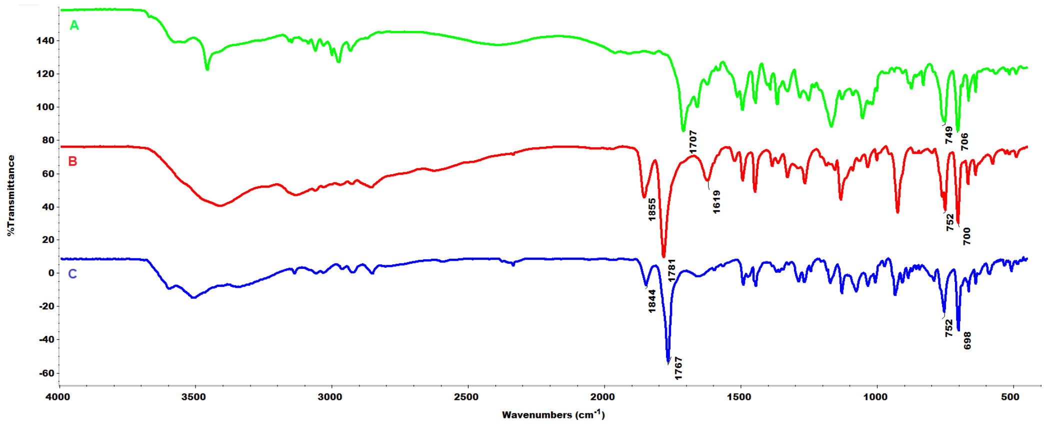

Figure S2. FT-IR spectra (A): of the precursor compound of BOC-His(*Trt*)-OH, (B): after 2.5 hours of reaction with $SOCl_2$, (C) final N^{im}-*Trityl*-L-His-NCA.

Synthesis of S-tert-Butyl mercapto-L-Cysteine N-Carboxy Anhydride (tBM-L-Cys-NCA)

The *N*-carboxy anhydride of *t*BM-L-Cys was synthesized in a similar way to a previously described process.[2] In a 500 mL three neck round-bottom flask 5.372 g of H-Cys(*St*Bu)-OH (25.663 mmol) were added and dried overnight under vacuum. Then, 120 mL of EtOAc were distilled in the flask, forming a white suspension.

At room temperature and under continuous argon flow, 9.22 mL (R)-(+)-limonene (56.986 mmol) were added in the reaction mixture. Then, the solution was heated gradually up to 50 °C and 5.3 g of triphosgene (17.894 mmol) diluted in 40 mL of distilled EtOAc were added dropwise over a period of 40 min. After the completion of the addition of triphosgene, the reaction mixture was stirred at 80 °C, in reflux, for 2,5 hours. Finally, the clear yellowish solution was concentrated at 40 mL and it was poured into 400 mL of cold hexane, followed by precipitation of *t*BM-L-Cys-NCA. Then, the solid was filtered, transferred to flask and finally dried in HVL. A second recrystallization was occurred with a mixture of solvent/non-solvent EtAc–Hexane (1:10) and the white solid precipitate *t*BM-L-Cys-NCA was isolated by filtration. Finally, *t*BM-L-Cys-NCA was dried under vacuum overnight and transferred into a glove box to afford 4.4 g (18.698 mmol, 73% yield). The purity of *t*BM-L-Cys-NCA was confirmed by ^{1}H NMR and FT-IR spectroscopy (Figure S3, S4). ^{1}H-NMR (600 MHz, $CDCl_3$, δ, ppm): 1.25–1.47 (c: 9H, $(\mathbf{CH_3})_3$–C–), 2.90–3.22 (b: 2H, –$\mathbf{CH_2}$–), 4.69–4.71 (a: 1H, –**CH**– of the NCA ring), 6.41 (d: 1H, –N**H**– of the NCA ring) (**Figure S3**). FT-IR (KBr, thin film): ~3200 cm^{-1} (*υ* -N-H, s), ~2855-2962 cm^{-1} (*υ* CH_2-S and CH_3 of *S*-*tert*-butyl group), ~686 cm^{-1}(*ν* S-S, s), 1535-1675 cm^{-1} (*υ* C=O and -NH_2, of precursor amino acid), 1828 cm^{-1} and 1855 cm^{-1} (*υ* C=O, s) **(Figure S4)**. The synthesis reaction of *t*BM-L-Cys-NCA is shown in **Scheme S2**.

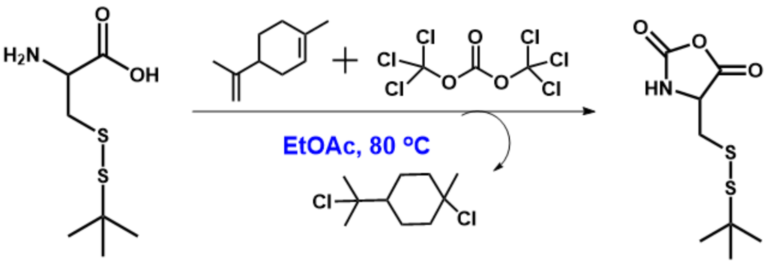

Scheme S2. The reaction synthesis of *t*BM-L-Cys-NCA.

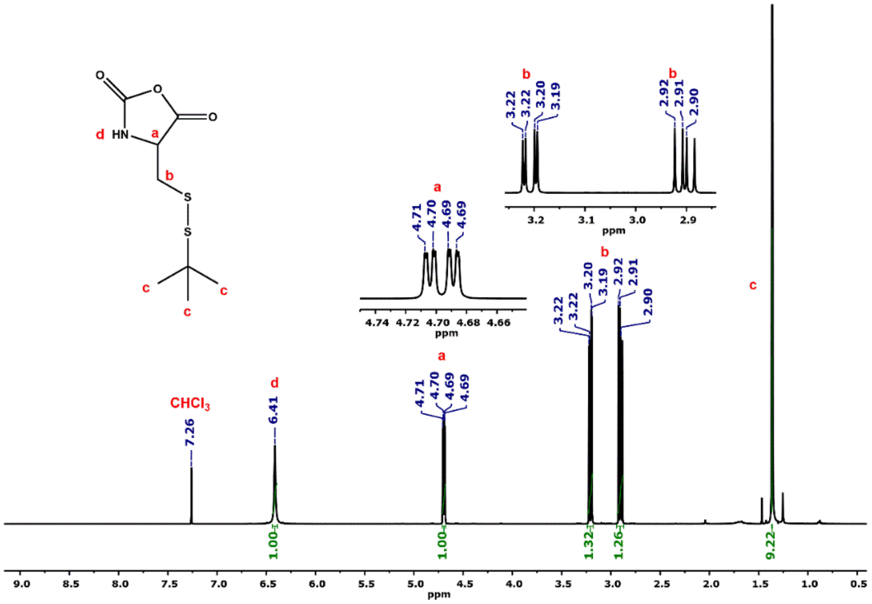

Figure S3. ^{1}H-NMR spectrum of the final *t*BM-L-Cys-NCA in $CDCl_3$.

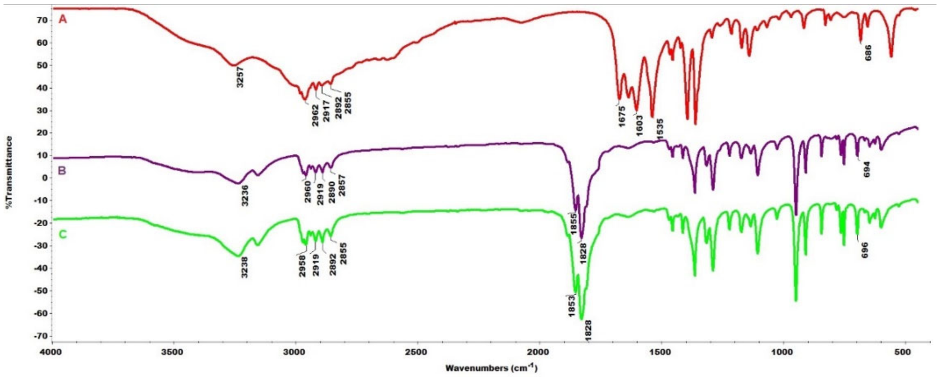

Figure S4. FT-IR spectra (A): of the precursor compound of H-Cys(S*t*Bu)-OH, (B): after 45 min of reaction with triphosgene, (C) final *t*BM-L-Cys-NCA.

Synthesis of Sarcosine N-Carboxy Anhydride (Sar-NCA)

The synthesis of the Sar-NCA was achieved by following previously reported procedures.[1-3] In a three-neck round bottom flask 10 g (112 mmol) of pulverized sarcosine were added and left drying overnight under high vacuum. Then 250 mL THF were distilled in the flask and 25 mL (145.6 mmol) of (+)(−) limonene were added giving a suspension. Subsequently the reaction mixture was heated at 50 °C and 11 g (37.3 mmol) of equimolar amount of triphosgene were added. The temperature of the reaction was further raised up to 70 °C. The reaction lasted about 1,5 hour and then the solution became clear. After complete dissolution, the reaction flask was attached to HVL, the solvent was distilled off and a brown solid was obtained and dried overnight under vacuum. The next day a sublimation process was conducted for the purification of the SAR-NCA. The procedure took place at 60 °C with the use of a custom made cold-finger glass apparatus under HV leading to the formation of SAR-NCA crystals. The same process was repeated once again and finally the desired product of SAR-NCA was collected and transferred into a glove box to afford 8.8 g (76.5 mmol, 68 %). The purity of SAR-NCA was confirmed by ^{1}H NMR and FT-IR spectroscopy (**Figure S5**, **S6**). ^{1}H-NMR (300 MHz, $CDCl_3$, δ, ppm): 3.02 (a: 3H, N-CH_3), 4.12 (b: 2H, N-CH_2-CO of the NCA ring). FT-IR (KBr, thin film): 1854, 1767 cm^{-1} (v C=O, s). The reaction synthesis of SAR-NCA is shown in **Scheme S3**.

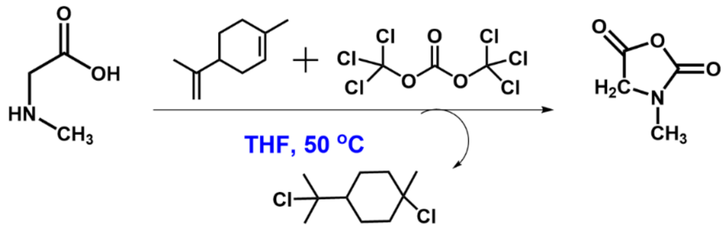

Scheme S3. The synthesis reaction of Sar-NCA.

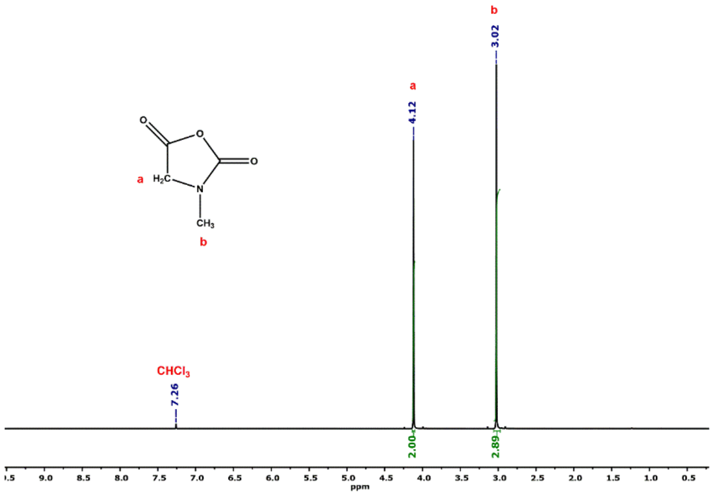

Figure S5. ^{1}H-NMR spectrum of the final Sar-NCA in $CDCl_3$.

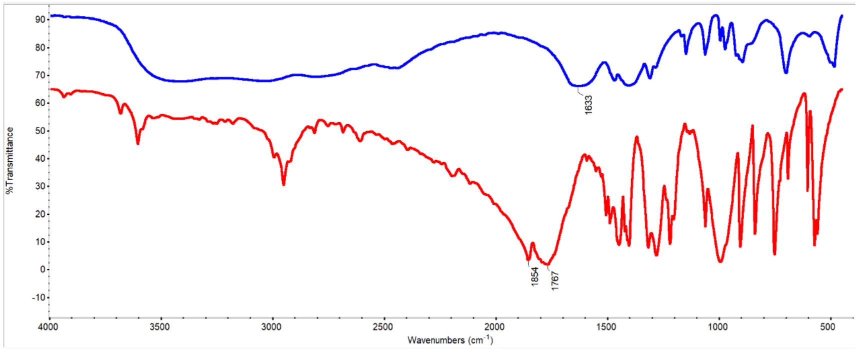

Figure S6. FT-IR spectra (A): of the precursor compound of Sarcosine, (B): final Sar-NCA.

Synthesis of the polymers

The synthesis of a series of hybrid polypeptide copolymers based on PHis and PCys was achieved through a ring-opening polymerization (ROP) process[4-6] of the corresponding *N*-carboxy anhydrides, using an amino end-functionalized poly(ethylene oxide) (*m*-PEO-NH_2) macroinitiator, with molecular weight 10.0×10^3 g mol^{-1}. More precisely, three different sets of polymers were synthesized, with two different molar ratios of monomers of L-histidine and L-cysteine. The difference relies, mainly, on the position of the building blocks, so in the first series two block copolymers of the type PCysX-PHis were constructed, the second set contains the type of PHis-PCysX while the last includes the random copolymers PCysXCOPHis. Each of the set was composed of two copolymers with different molar ratios of cysteine to histidine, 8.6% and 17.7%.

High vacuum techniques[7-11] were employed not only for the preparation of the *N*-carboxy anhydrides of α-amino acids, but also for the synthesis of well-defined polymers.

In order to ensure the high purity of the system and follow the guidelines of the high vacuum techniques, custom-made glass reactors were utilized, comprised of break-seals, high vacuum stopcock, glass-covered magnets and constrictions for the addition of reagents. The procedure followed for the synthesis of the PEO-*b*-[P(Cys)-*co*-P(His)] polymers is described below and the reactions conducted in the process are presented in Scheme S1. In case of PCysX-PHis as well as PHis-PCysX copolypeptides, the sequential monomer addition was followed, while at the PCysXCOPHis the two monomers were polymerized together. Since the PEO blocks were equal for all the polymers, the code of the hybrid polypeptides was defined by the order of the blocks as well as the monomeric units of L-cysteine, therefore the abbreviation PHis-PCys5 refers to the triblock *m*PEO_{227}-*b*-$P(His)_{40}$-*b*-$P(Cys)_5$ and PCys10-PHis for *m*PEO_{227}-*b*-$P(Cys)_{10}$-*b*-$P(His)_{35}$, while in case of the *m*PEO_{227}-*b*-[$P(Cys)_5$-*co*-$P(His)_{40}$ where the polypeptidic block is composed of randomly distributed peptides will be mentioned PCys5COPHis.

With the use of glass blowing techniques, a specially designed polymerization reactor was constructed composed of a 250 mL polymerization flask and two ampoules with break–seals, for each one of the monomers. Firstly, 0.6 g of *m*PEO-NH_2 with molecular weight 10.0×10^3 g mol^{-1} (0.06 mmol) were added in the flask and left overnight for drying in high vacuum line. The amount of the macroinitiator was kept constant for each of the six synthesized polymers. The next day, 20 mL of purified benzene were distilled in order to azeotropically remove traces of water. The solution was stirred for two hours and then the solvent was distilled off and the macroinitiator was left to dry overnight. The following day, fractional distillation of 40 mL DMF was occurred in order to dissolve the macroinitiator. Afterwards, the apparatus was detached from the HVL and connected again via the one side ampoule in order to remove traces of water by flame–drying, as NCAs are very sensitive to moisture. Then, the glass reactor was inserted in the glove box, where the monomers are kept under argon inert atmosphere and the addition of the first *N*-carboxy anhydride was conducted. In the case of the polymers of the type of PCys-PHis, the *S*-*tert*-Butyl-mercapto-L-Cysteine NCA was added first, while the *N^{im}-Trityl*-L-His NCA was used as the first monomer in the polymerization of the type of PHis-PCysX copolymers. As far as the random copolymers PCysXCOPHis are concerned, both NCAs were added together in the same ampoule. Subsequently, the ampoule was attached to the HVL and was degassed in order to remove the argon gas. Purified DMF (5 mL) was directly distilled into the ampoule with the monomer and then the apparatus was removed from the HVL through heat sealing. In the aftermath, the monomer was completely dissolved in DMF and the break–seal of the ampoule was broken, leading to the complete fusion of the two solutions, and consequently to the initiation of the polymerization. The reactor was connected again to the HVL and the reaction mixture left under stirring. The ring opening polymerization reaction was accompanied by the production of carbon dioxide, therefore, an occasional degassing process was required in order for the reaction to be continued. In addition, the progress of the polymerization was observed by extracting a small aliquot of the reaction mixture inside the glove box and analyzing it, with the use of FT-IR spectroscopy. The absence of the characteristic peaks of the monomer at almost 1786 cm^{-1} and 1821 cm^{-1} as well as the existence of the peak at 1650 cm^{-1} due to the peptide bond revealed either the consumption of the first monomer, in the case of the triblock terpolymers, or the completion of the reaction in the case of the random copolymers. Moreover, as far as the block copolymers are concerned, after the reaction of the first monomer, the second *N*-carboxy anhydride was added in the other ampoule of the apparatus, following exactly the same procedure as described above.

Deprotection of Polypeptides

Deprotection of Poly(N^{im}-Trityl-L-Histidine) Blocks

In a typical procedure (Scheme S2), the fully protected polymer was suspended in 20 mL CH_2Cl_2 and left stirring for 1 hour. Then, 12 mL of trifluoroacetic acid (TFA) were

added to the reaction mixture and the solution turned from yellowish and turbid to bright yellow and clear, indicating the deprotection of the *trityl* groups of poly(*N*^{im}-*Trityl*-L-Histidine) chains. The reaction was maintained at room temperature for 1 hour and then 0.9 mL of triisopropylsilane $(iPr)_3SiH$ were added (3:1 molar ratio with respect to the number of histidine monomeric units), in order to bind the cleaved *trityl* groups. At the equilibrium point the solution turned from bright yellow to colorless. Afterwards, all the solvents were distilled off in HVL and the received solid was dried overnight under vacuum.

The next day the partially deprotected polymer was dissolved in 30 mL of Milli-Q water, and then it was filtered through a borosilicate funnel Por 3, in order to separate the insoluble *trityl* groups from the soluble deprotected polymer (filtrate). Then the pH of the filtrate was adjusted to pH = 5.9 with the addition of dilute K_2CO_3 10% solution and transferred into a dialysis bag (MWCO=3500 Da, Fisher Scientific). After that, it was dialyzed against 2 L of Milli-Q water with pH ~ 3 (adjusted with a dilute solution of HCl) for two times, five times against 2 L of Milli-Q water with pH ~ 10 (adjusted with a dilute solution of NaOH) and two times with pure Milli-Q water. The whole dialysis procedure lasted for 6 days until the pH value was close to neutral. Finally, the polymer solution was freeze dried and characterized by SEC, ^{1}H-NMR and FT-IR spectroscopy.

Deprotection of Poly(L-Cysteine)

The partially deprotected polymer, as far as the *trityl* groups are concerned, was dissolved in 40 mL DMF (Scheme S3). Then excess of dithiothreitol (DTT) (9:1 molar ratio with respect to the number of cysteine monomeric units) was added and the reaction mixture was left stirring for seven days, at 60 °C and under argon atmosphere. Subsequently, the solution was precipitated in 500 mL of $(Et)_2O$, and the product was isolated by vacuum filtration in a Buchner funnel using a PTFE hydrophobic filter of 0.45 μm. All the procedures above were conducted under argon flow. The obtained full deprotected polymer was dried overnight in HVL and was preserved in the glove box for further use, in order to avoid the undesired oxidation of the free thiol groups. Finally, it was characterized by SEC, ^{1}H-NMR and FT-IR spectroscopy.

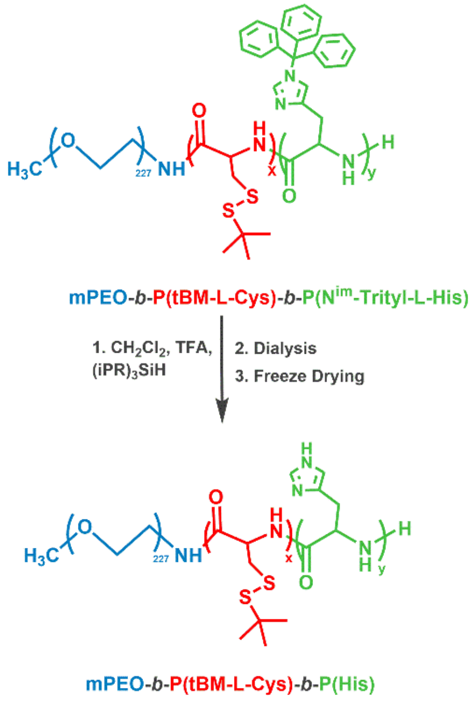

Scheme S4. Deprotection Reaction of the poly(*N*^{im}-*Trityl*-L-Histidine) of the polymers of the type PEO-*b*-P(*N*^{im}-*Trityl*-L-His)-*b*-P(*t*BM-L-Cys).

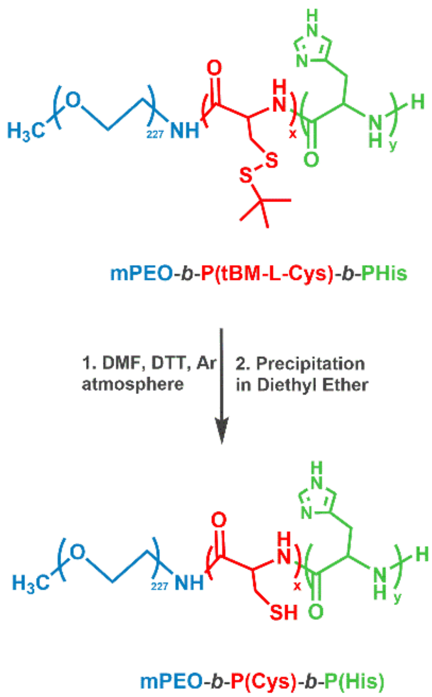

Scheme S5. Deprotection Reaction of the poly(*t*BM-L-Cysteine) of the polymers of the type PEO-*b*-P(*t*BM-L-Cys)-*b*-P(His).

Molecular Characterization of the Polymers

Molecular Characterization of PCys10-PHis

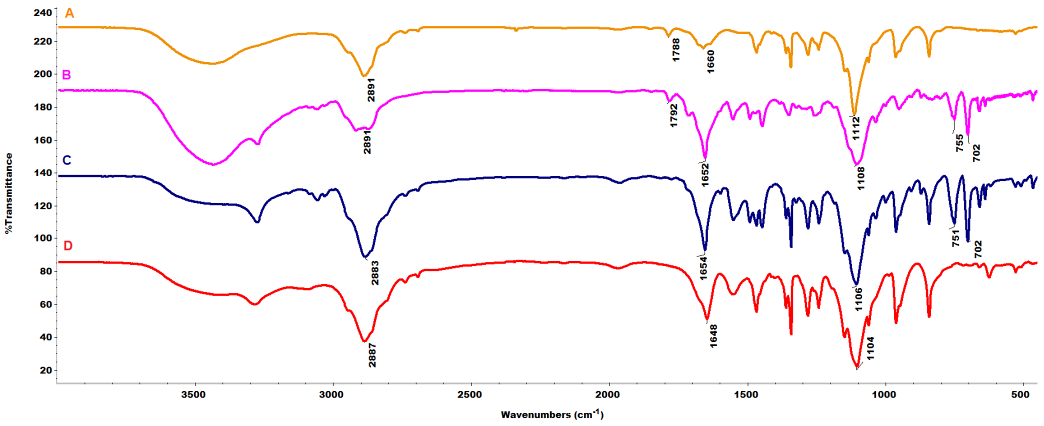

Figure S7. FT - IR spectra of A. *m*PEO$_{227}$-*b*-P(*t*BM-L-Cys)$_{10}$, B. *m*PEO$_{227}$-*b*-P(*t*BM-L-Cys)$_{10}$-*b*-P(N^{im}-*Trityl*-L-His)$_{35}$ 10 days after the addition of NCA His, C. *m*PEO$_{227}$-*b*-P(*t*BM-L-Cys)$_{10}$-*b*-P(N^{im}-*Trityl*-L-His)$_{35}$, D. fully deprotected PCys10-PHis.

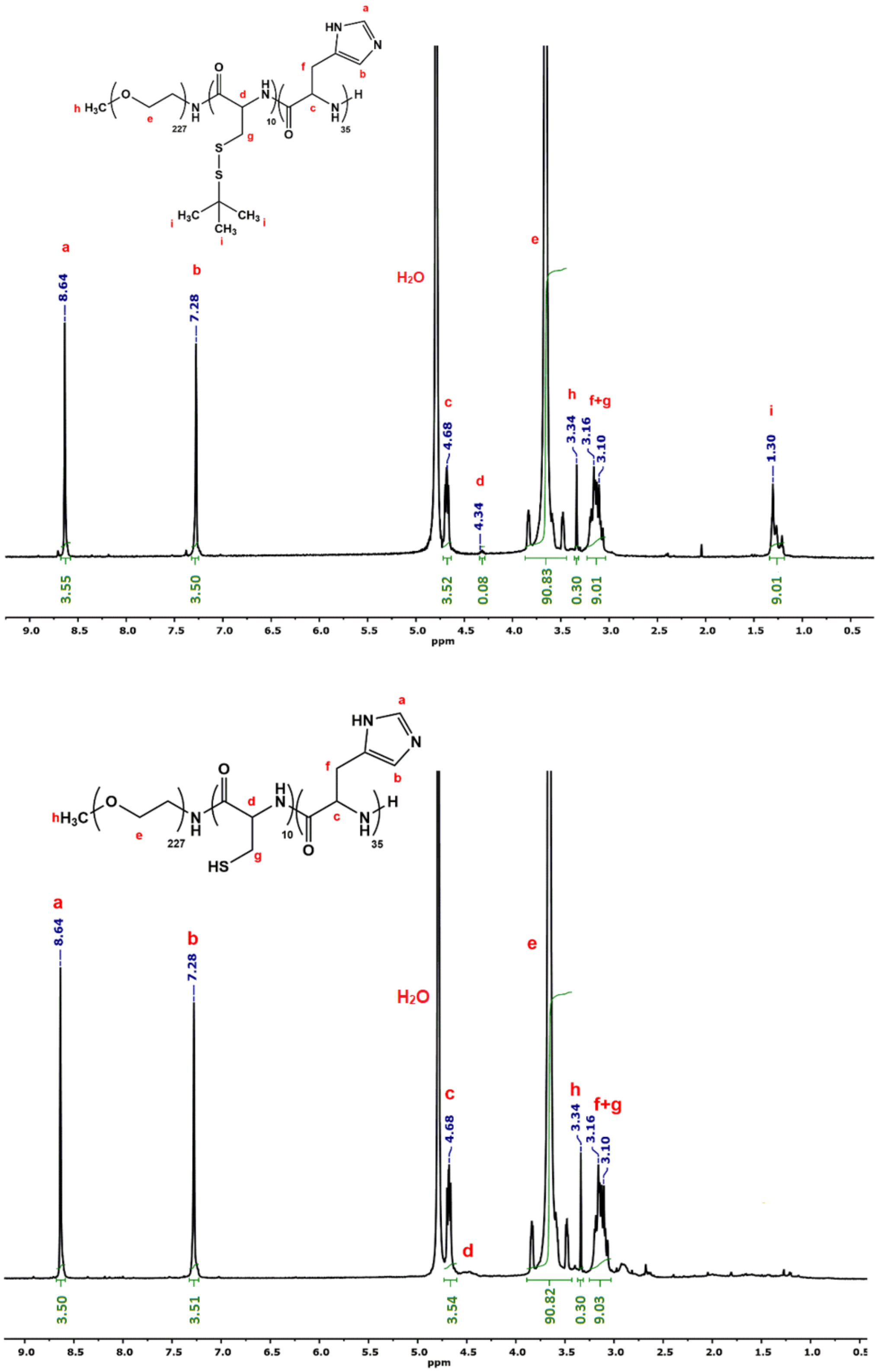

Figure S8. ^{1}H-NMR spectra of the Histidine – deprotected *m*PEO$_{227}$-*b*-P(*t*BM-L-Cys)$_{10}$-*b*-P(His)$_{35}$ and the fully deprotected PCys10-PHis, in D_2O / DCl 1%.

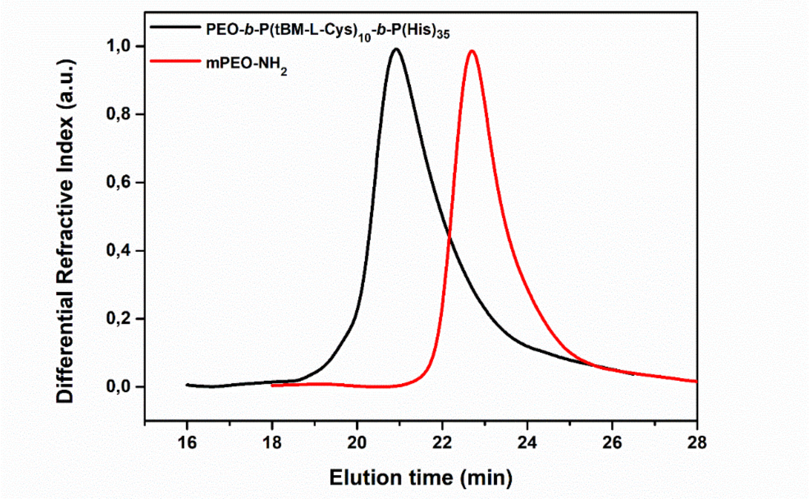

Figure S9. SEC eluogram of the *m*PEO-NH_2 and *m*PEO_{227}-*b*-P(*t*BM-L-Cys)$_{10}$-*b*-P(His)$_{35}$ in H_2O / TFA.

Molecular Characterization of PHis-PCys5

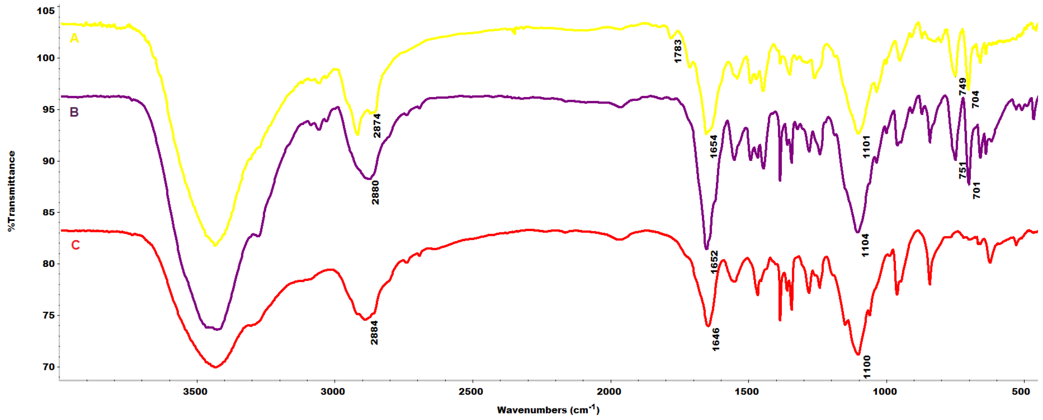

Figure S10. FT - IR spectra of A. *m*PEO_{227}-*b*-P(*N^{im}-Trityl*-L-His)$_{40}$, B. *m*PEO_{227}-*b*-P(*N^{im}-Trityl*-L-His)$_{40}$-*b*-P(*t*BM-L-Cys)$_5$, C. fully deprotected PHis-PCys5.

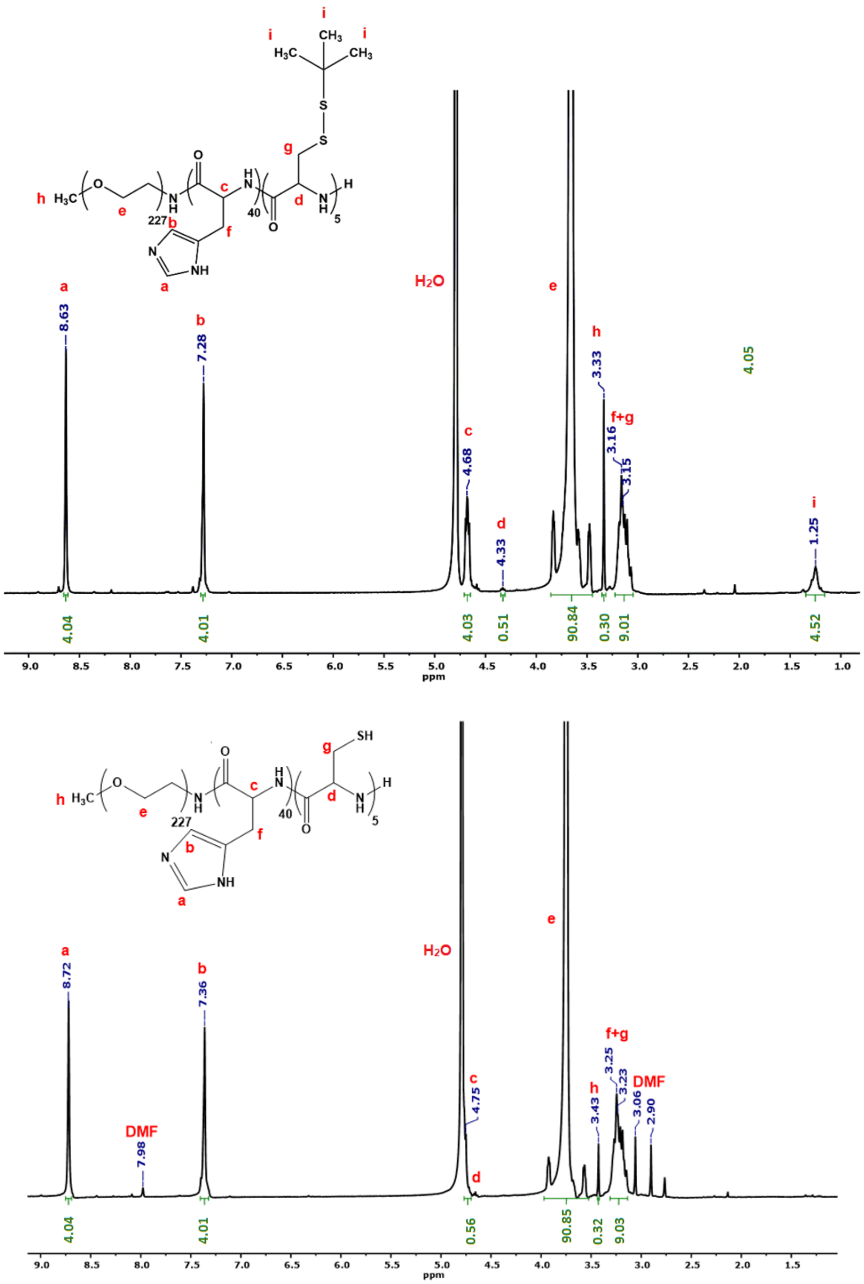

Figure S11. ^{1}H-NMR spectra of the Histidine – deprotected *m*PEO$_{227}$-*b*-P(His)$_{40}$-*b*-P(*t*BM-L-Cys)$_5$ and the fully deprotected PHis-PCys5, in D_2O / DCl 1%.

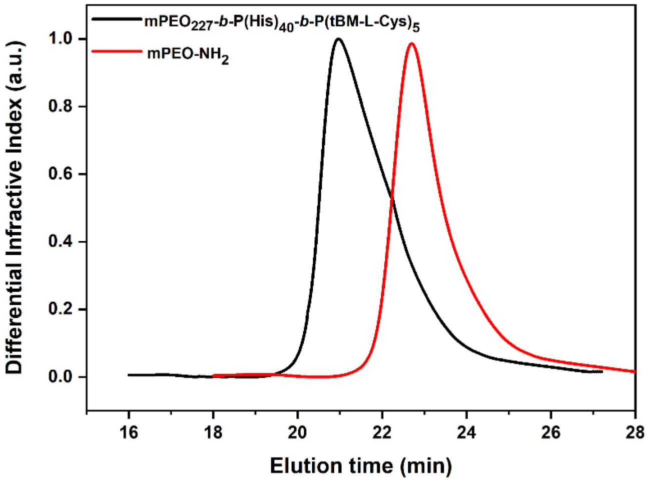

Figure S12. SEC eluogram of the *m*PEO$_{227}$-*b*-P(His)$_{40}$-*b*-P(*t*BM-L-Cys)$_5$ in H_2O / TFA.

Molecular Characterization of PHis-PCys10

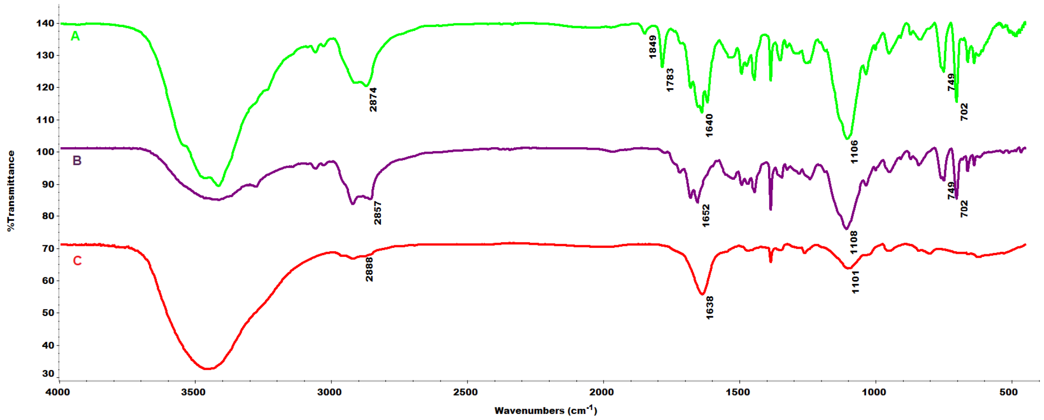

Figure S13. FT - IR spectra of A. *m*PEO$_{227}$-*b*-P(*N*^{im}-*Trityl*-L-His)$_{35}$, B. *m*PEO$_{227}$-*b*-P(*N*^{im}-*Trityl*-L-His)$_{35}$-*b*-P(*t*BM-L-Cys)$_{10}$, C. fully deprotected PHis-PCys10.

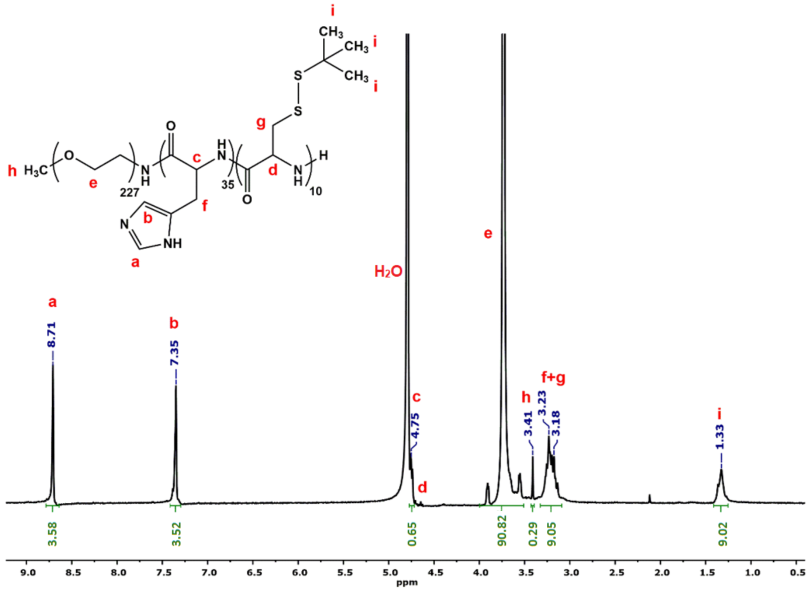

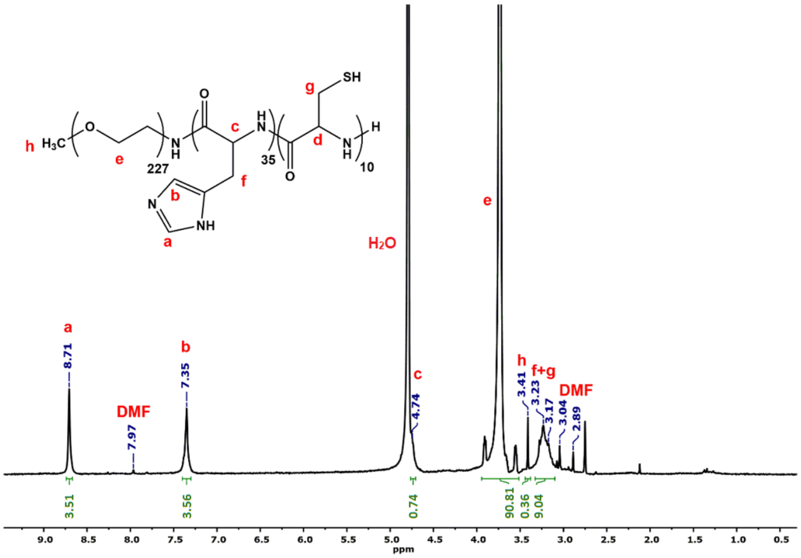

Figure S14. ^{1}H-NMR spectra of the Histidine – deprotected *m*PEO227-*b*-P(His)35-*b*-P(*t*BM-L-Cys)10 and the fully deprotected PHis-PCys10, in D_2O / DCl 1%.

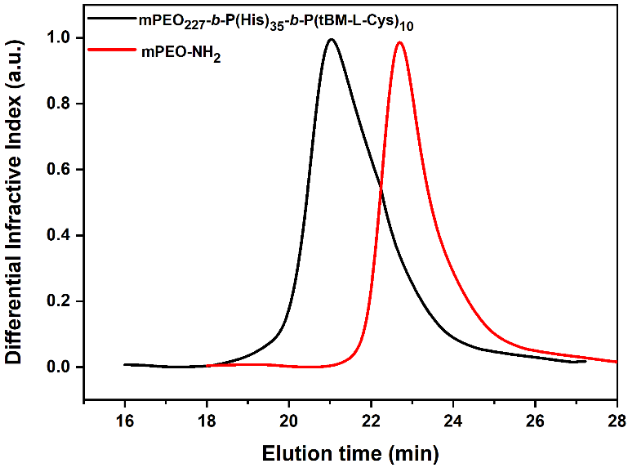

Figure S15. SEC eluogram of the *m*PEO227-*b*-P(His)35-*b*-P(*t*BM-L-Cys)10 in H_2O / TFA.

Molecular Characterization of PCys5COPHis

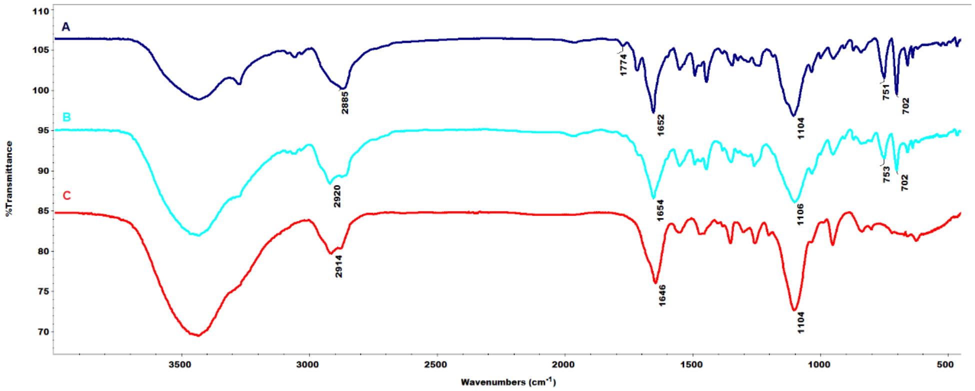

Figure S16. FT - IR spectra of A. mPEO$_{227}$-b-[P(tBM-L-Cys)$_5$-co-P(N^{im}-$Trityl$-L-His)$_{40}$] 5 days after the initiation of polymerization process. B. mPEO$_{227}$-b-[P(tBM-L-Cys)$_5$-co-P(N^{im}-$Trityl$-L-His)$_{40}$]. C. fully deprotected PCys5COPHis.

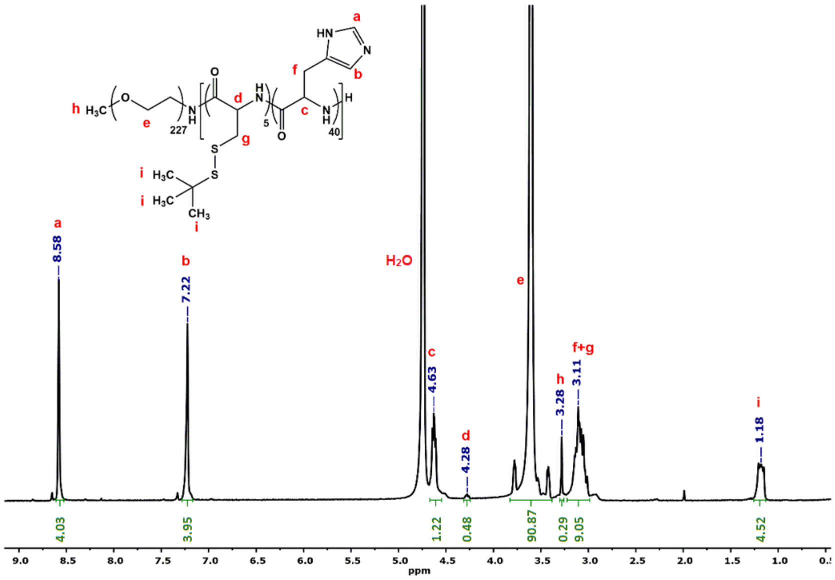

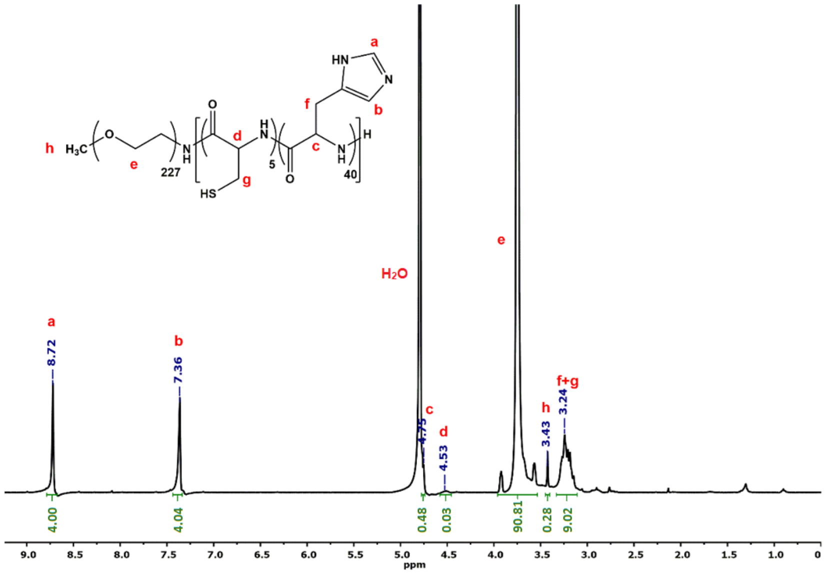

Figure S17. ^{1}H-NMR spectra of the Histidine – deprotected mPEO$_{227}$-b-[P(tBM-L-Cys)$_5$-co-P(His)$_{40}$] and the fully deprotected PCys5COPHis, in D_2O / DCl 1%.

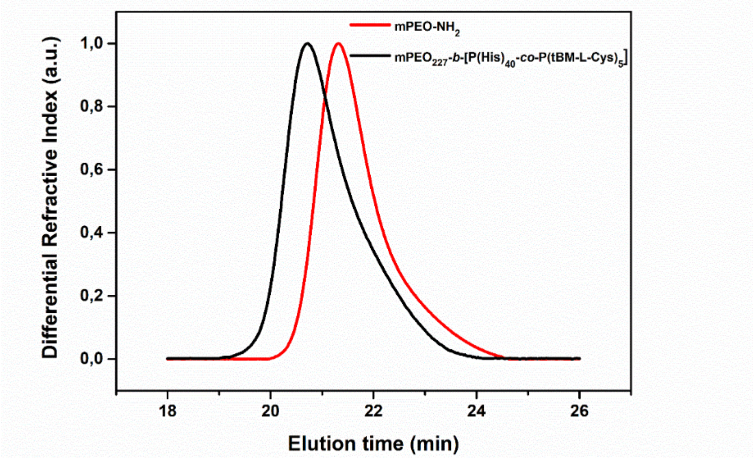

Figure S18. SEC eluogram of the *m*PEO-NH_2 and *m*PEO_{227}-*b*-[P(*t*BM-L-Cys)$_5$-*co*-P(His)$_{40}$] in H_2O / TFA.

Molecular Characterization of PCys10COPHis

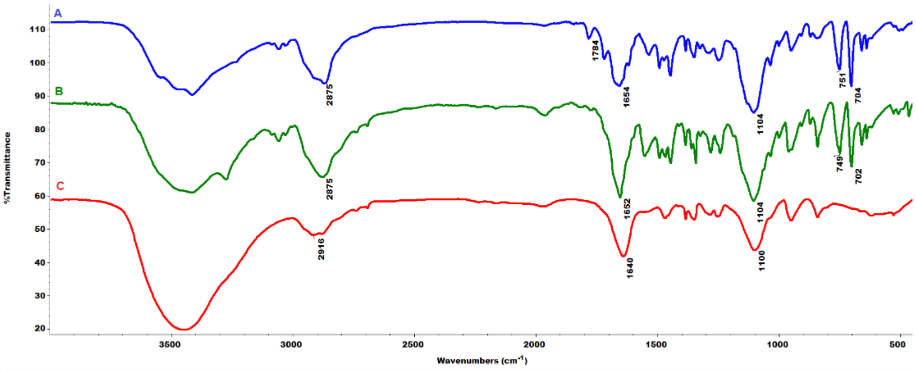

Figure S19. FT - IR spectra of A. *m*PEO_{227}-*b*-[P(*t*BM-L-Cys)$_{10}$-*co*-P(N^{im}-*Trityl*-L-His)$_{35}$] 5 days after the initiation of polymerization process, B. final *m*PEO_{227}-*b*-[P(*t*BM-L-Cys)$_{10}$-*co*-P(N^{im}-*Trityl*-L-His)$_{35}$], C. fully deprotected PCys10COPHis.

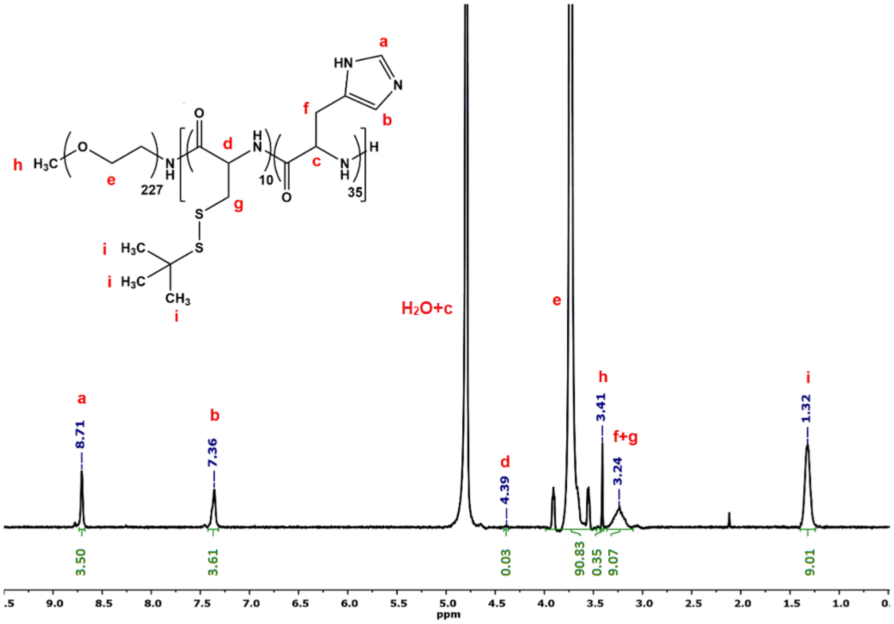

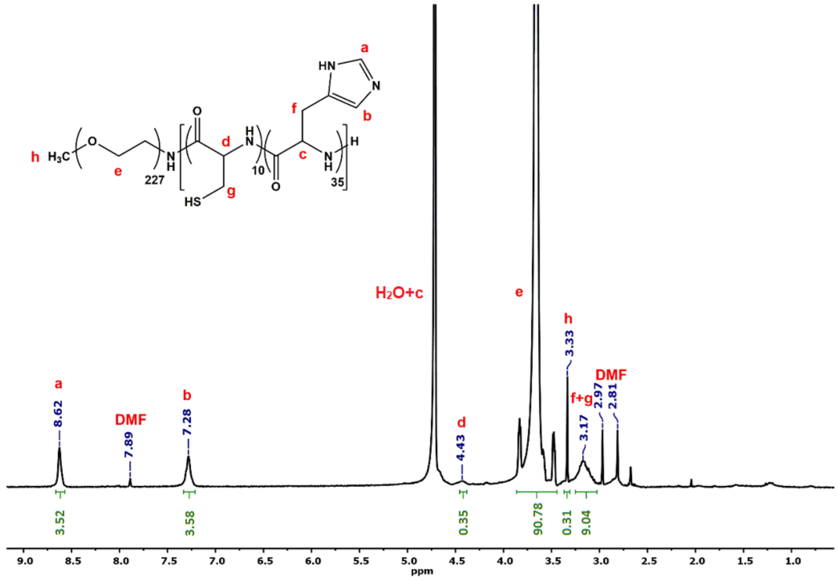

Figure S20. ^{1}H-NMR spectra of the Histidine – deprotected mPEO$_{227}$-*b*-[P(*t*BM-L-Cys)$_{10}$-*co*-P(His)$_{35}$] and the fully deprotected PCys10COPHis, in D_2O / DCl 1%.

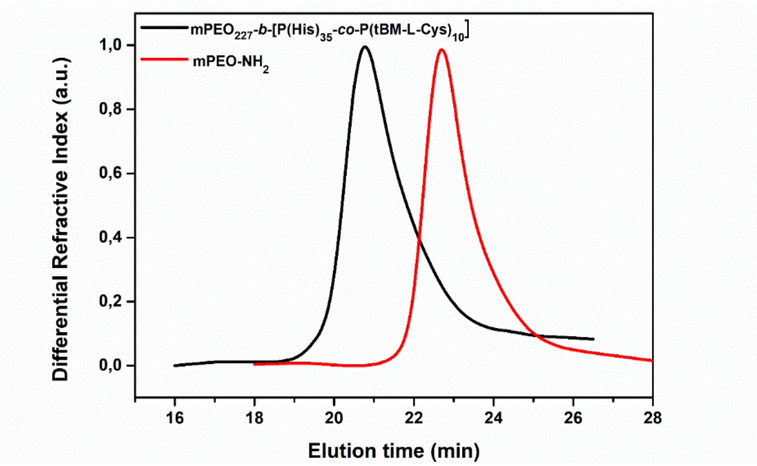

Figure S21. SEC eluogram of the mPEO-NH_2 and mPEO$_{227}$-*b*-[P(*t*BM-L-Cys)$_{10}$-*co*-P(His)$_{35}$] in H_2O / TFA.

Molecular Characterization of PCys5-PHis

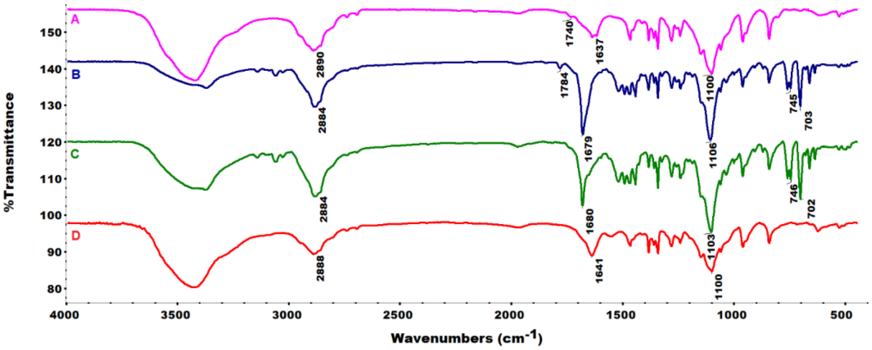

Figure S22. FT-IR spectrum of A. *m*PEO227-*b*-P(*t*BM-L-Cys)5, B. *m*PEO227-*b*-P(*t*BM-L-Cys)5-*b*-P(*N*^{im}-*Trityl*-L-His)40 14 days after the addition of *N*^{im}-*Trityl*-L-His NCA, C. *m*PEO227-*b*-P(*t*BM-L-Cys)5-*b*-P(*N*^{im}-*Trityl*-L-His)40, D. fully deprotected PCys5-PHis.

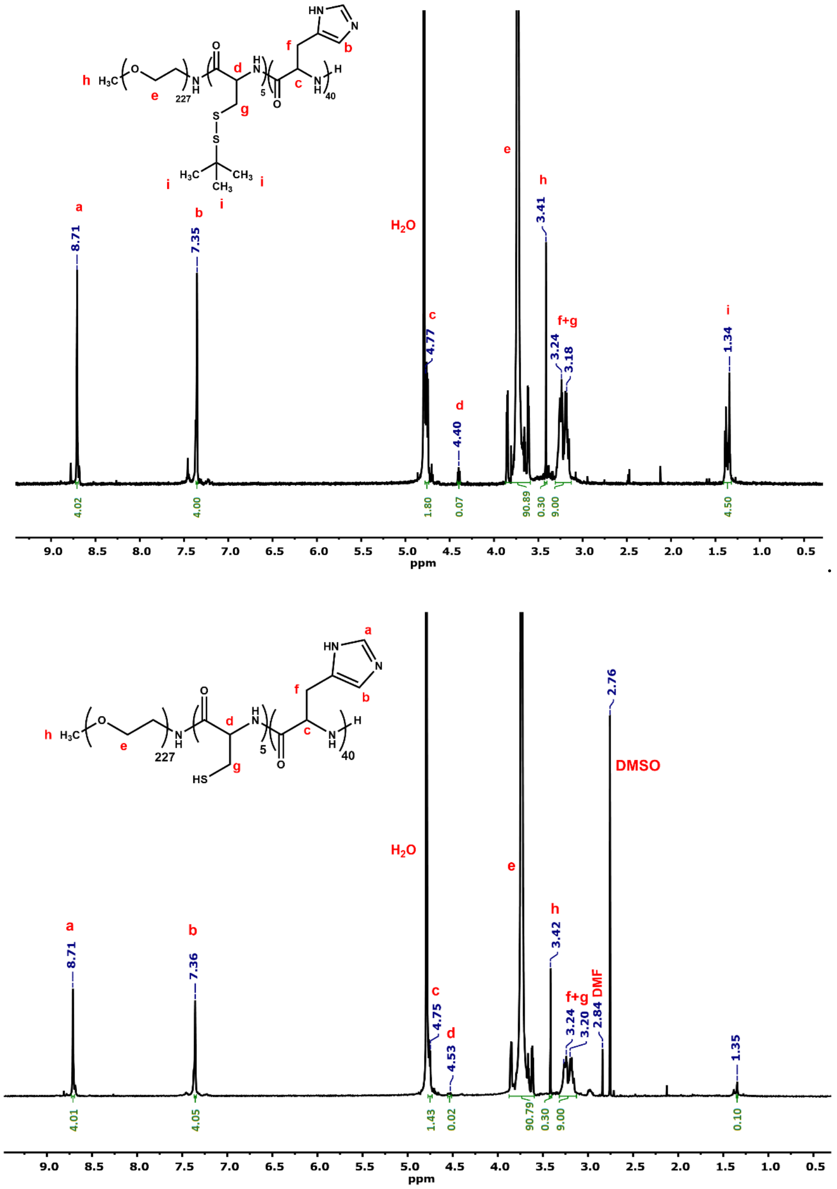

Figure S23. ^{1}H-NMR spectra of the histidine-deprotected *m*PEO227-*b*-P(*t*BM-L-Cys)5-*b*-P(His)40 and the fully deprotected PCys5-PHis, in D2O/DCL 1%.

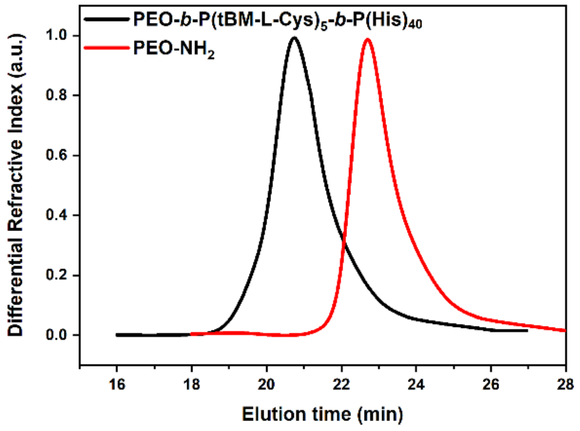

Figure S24. SEC chromatogram of the *m*PEO-NH_2 and *m*PEO_{227}-*b*-P(*t*BM-L-Cys)$_5$-*b*-P(His)$_{40}$ in H_2O/TFA.

Molecular Characterization of mPEO$_{227}$*-b-P(Sar)*$_{98}$*-b-P(Cys)*$_{30}$

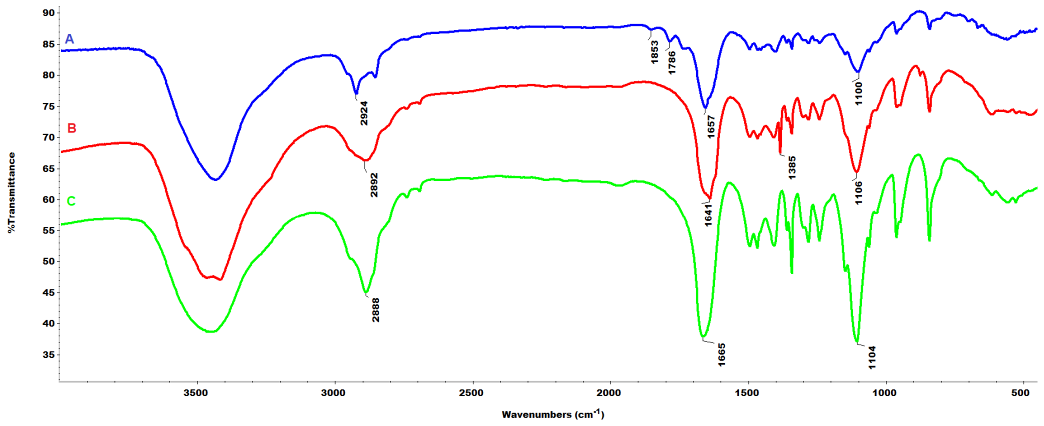

Figure S25. FT - IR spectra of A. *m*PEO_{227}-*b*-P(Sar)$_{98}$, B. *m*PEO_{227}-*b*-P(Sar)$_{98}$-*b*-P(*t*BM-L-Cys)$_{30}$, C. fully deprotected *m*PEO_{227}-*b*-P(Sar)$_{98}$-*b*-P(Cys)$_{30}$.

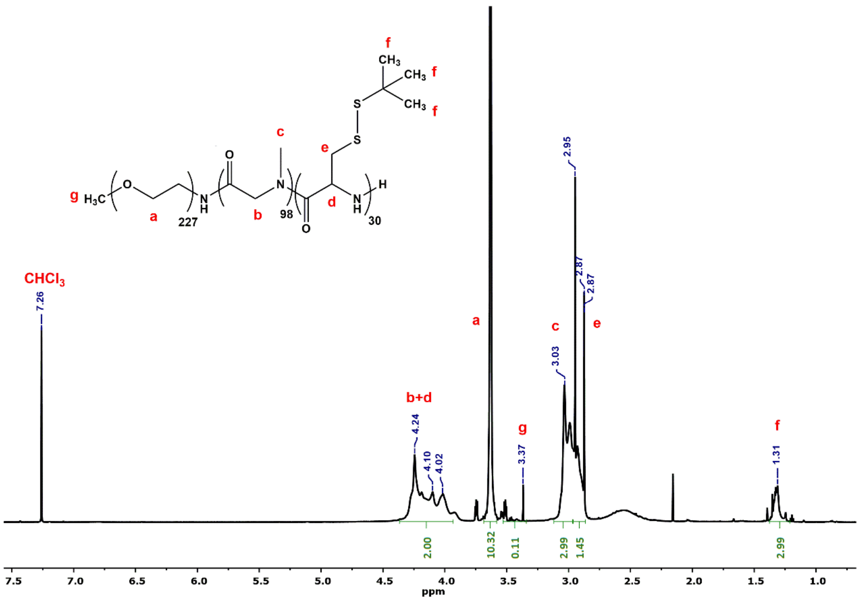

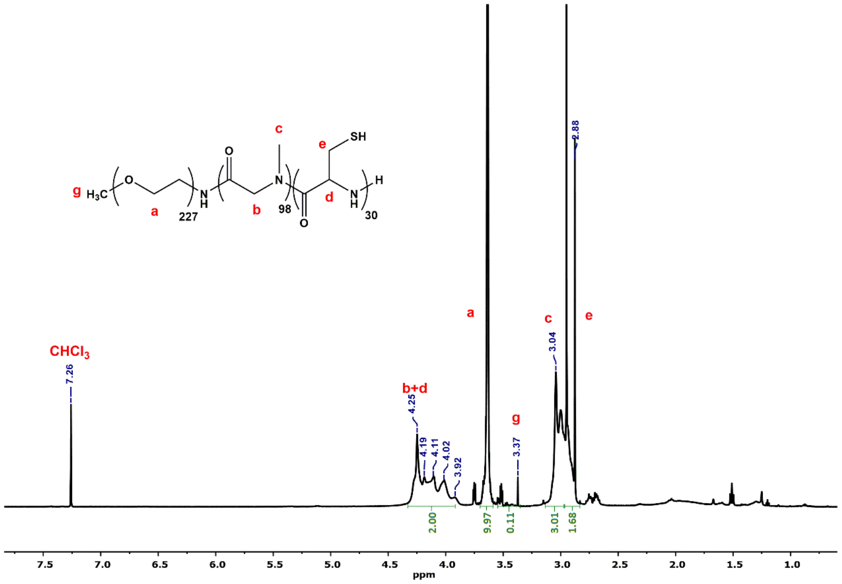

Figure S26. ^{1}H-NMR spectra of the protected *m*PEO$_{227}$-*b*-P(Sar)$_{98}$-*b*-P(*t*BM-L-Cys)$_{30}$ and the fully deprotected *m*PEO$_{227}$-*b*-P(Sar)$_{98}$-*b*-P(Cys)$_{30}$, in $CDCl_3$.

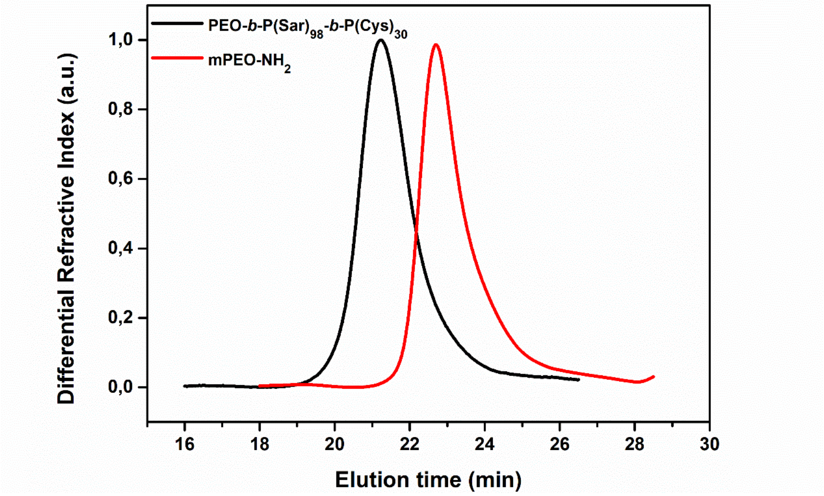

Figure S27. SEC eluogram of the *m*PEO-NH$_2$ and *m*PEO$_{227}$-*b*-P(Sar)$_{98}$-*b*-P(Cys)$_{30}$ in H_2O / TFA.

Molecular Characterization of mPEO$_{227}$-b-PHis$_{44}$

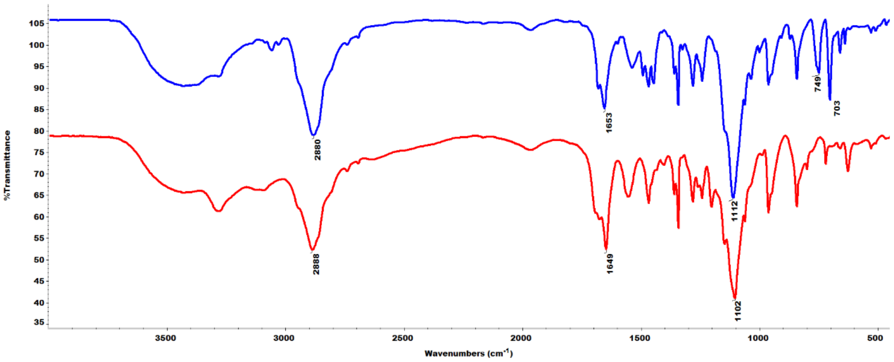

Figure S28. FT - IR spectra of A. protected *m*PEO$_{227}$-*b*-P(*N*^{im}-*Trityl*-L-His)$_{44}$, B. deprotected *m*PEO$_{227}$-*b*-P(His)$_{44}$.

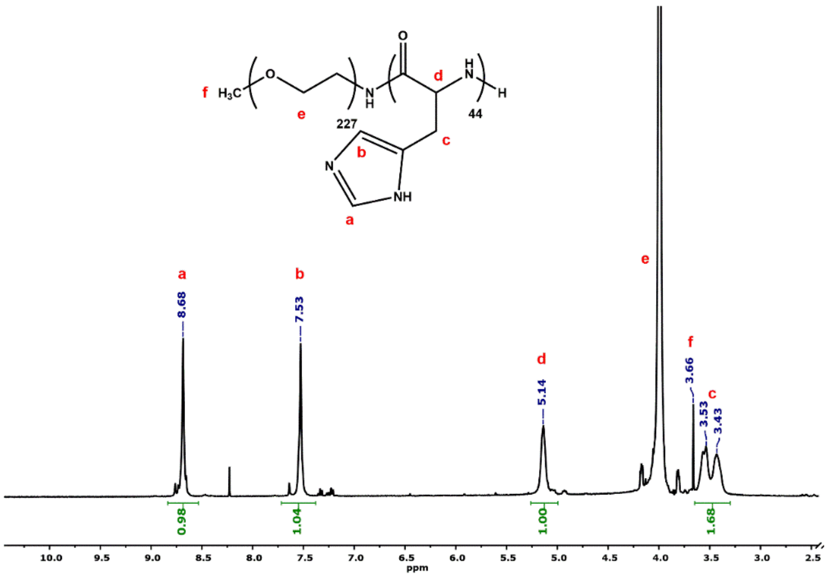

Figure S29. ^{1}H-NMR spectrum of the fully deprotected *m*PEO$_{227}$-*b*-P(His)$_{44}$, in TFA.

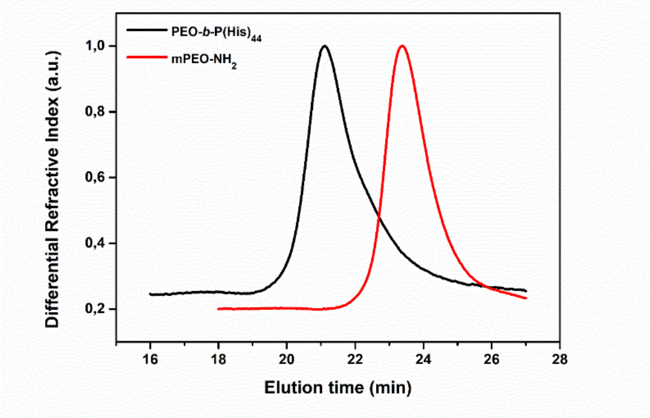

Figure S30. SEC eluogram of the *m*PEO-NH$_2$ and *m*PEO$_{227}$-*b*-P(His)$_{44}$ in H_2O / TFA.

Circular Dichroism Measurements of the polymers

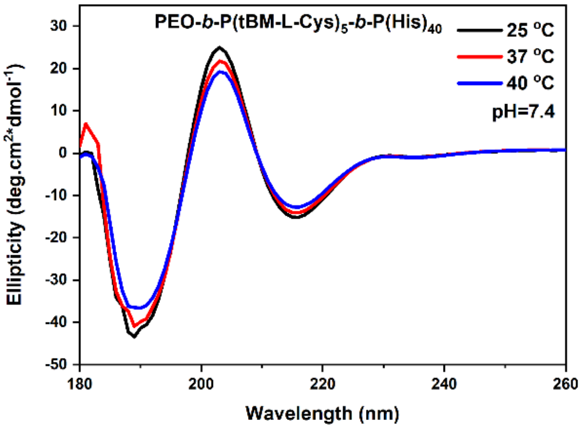

Figure S31. CD spectra of *m*PEO$_{227}$-*b*-P(*t*BM-L-Cys)$_5$-*b*-P(His)$_{40}$ at pH = 7.4, at different temperatures.

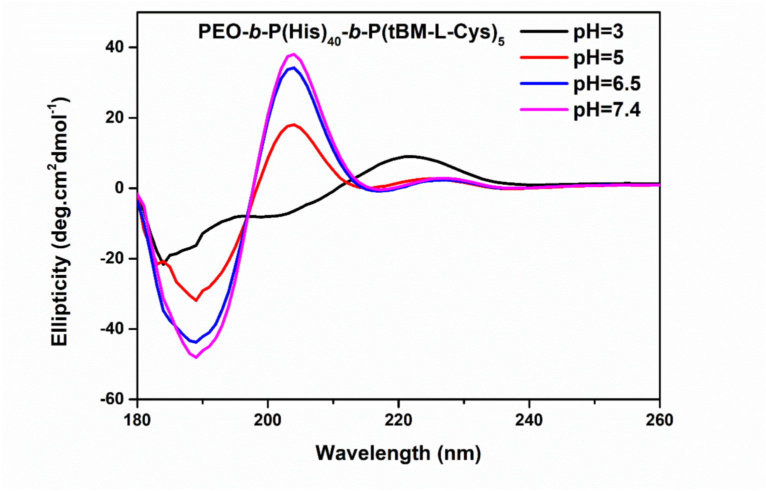

Figure S32. CD spectra of *m*PEO$_{227}$-*b*-P(His)$_{40}$-*b*-P(*t*BM-L-Cys)$_5$ at different pH, at 25 °C.

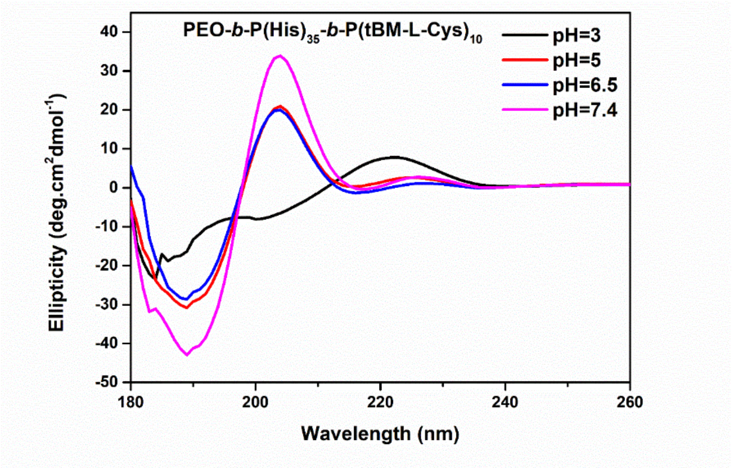

Figure S33. CD spectra of *m*PEO$_{227}$-*b*-P(His)$_{35}$-*b*-P(*t*BM-L-Cys)$_{10}$ at different pH, at 25 °C.

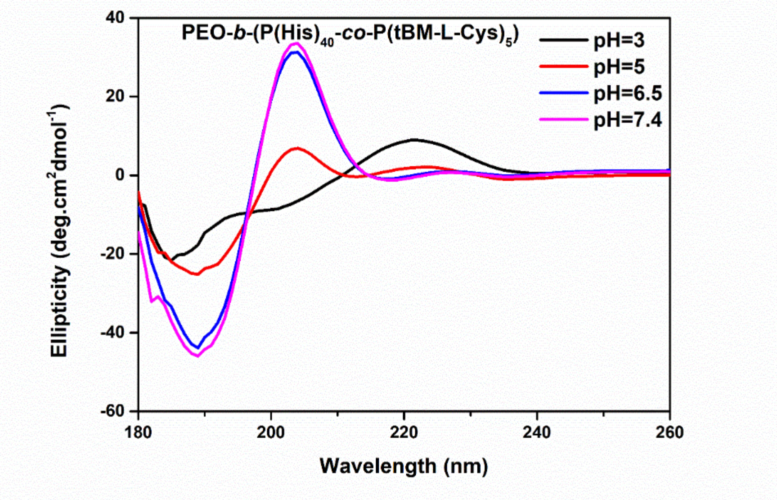

Figure S34. CD spectra of *m*PEO$_{227}$-*b*-[P(*t*BM-L-Cys)$_5$-*co*-P(His)$_{40}$] at different pH, at 25 °C.

DLS measurements of the empty nanoparticles

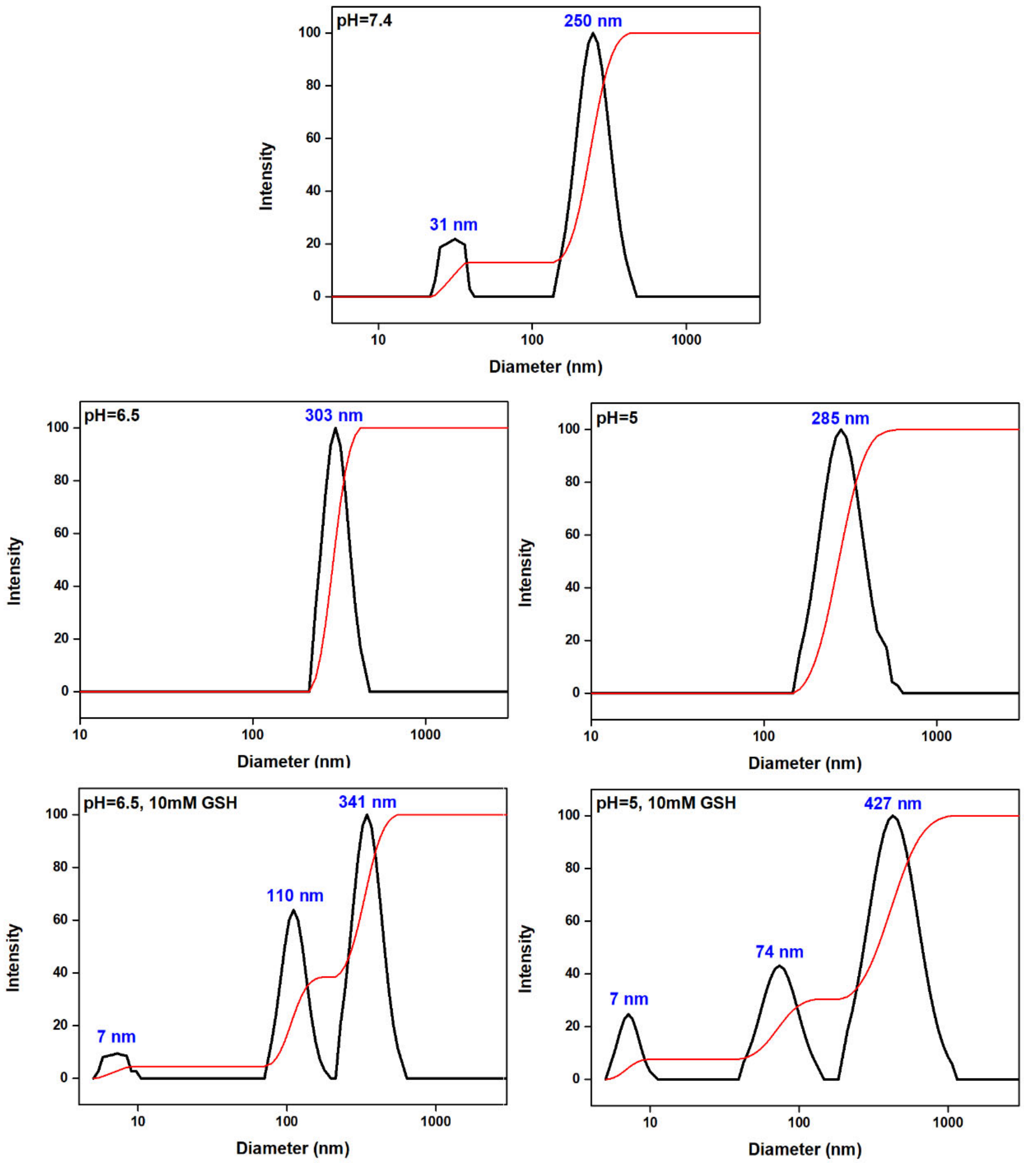

Figure S35. Schematic representation of the size dependence of the PCys5-PHis NPs from pH and GSH concentration, at 25 °C, at 90°.

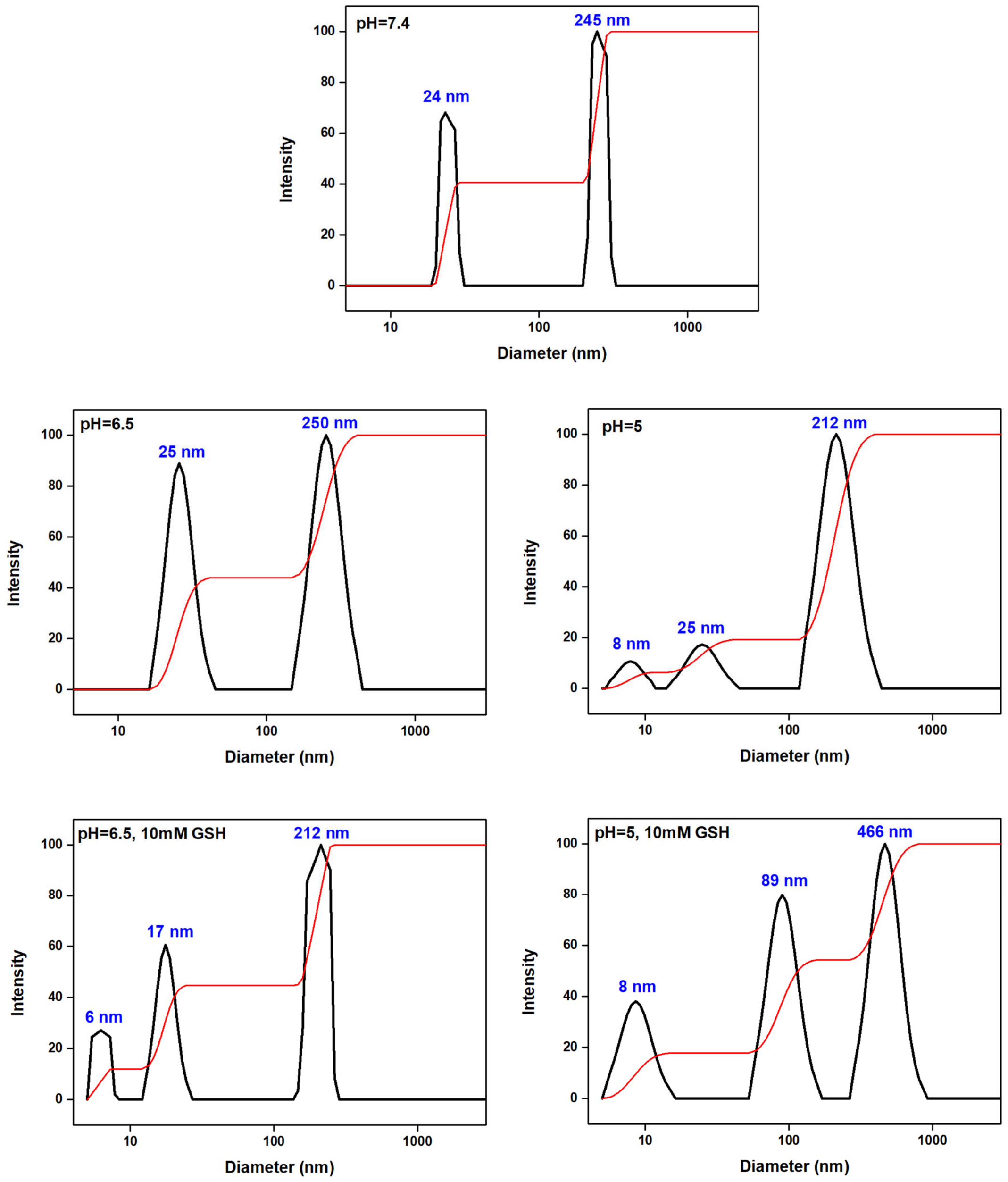

Figure S36. Schematic representation of the PHis-PCys5 NPs size dependence from pH and GSH concentration, at 25 °C, at 90°.

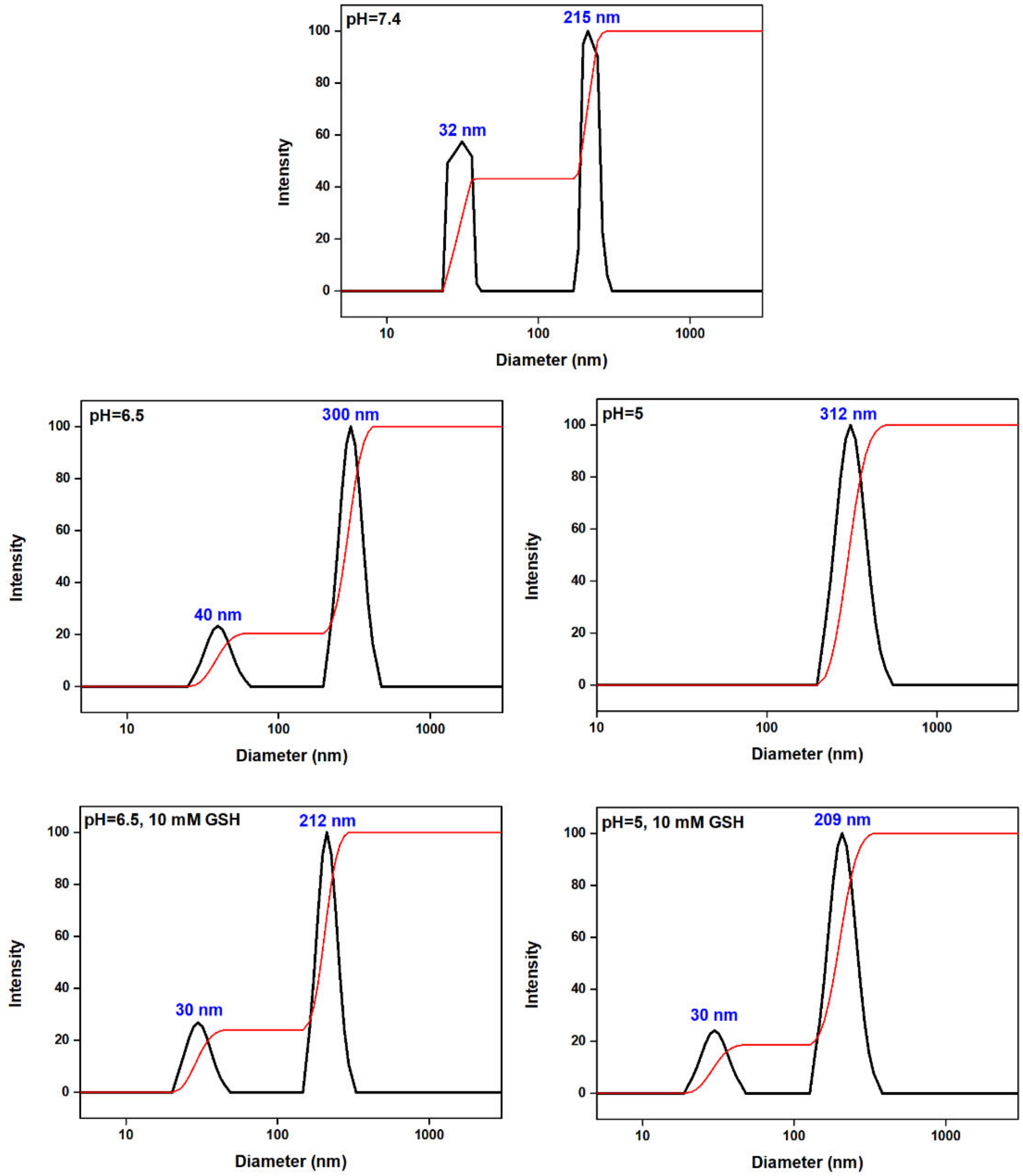

Figure S37. Schematic representation of the PHis-PCys10 NPs size dependence from pH and GSH concentration, at 25 °C, at 90°.

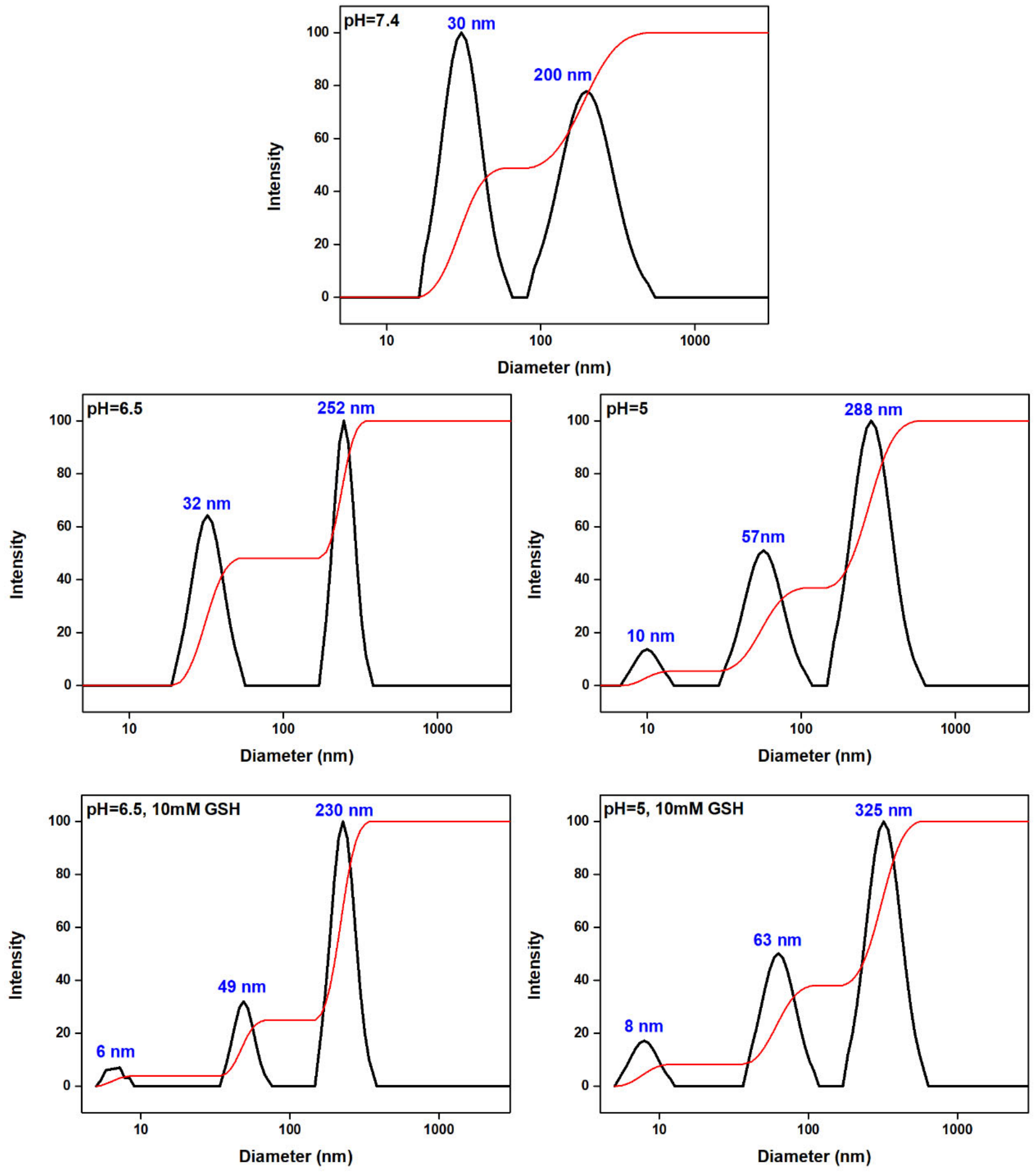

Figure S38. Schematic representation of the PCys5COPHis NPs size dependence from pH and GSH concentration, at 25 °C, at 90°.

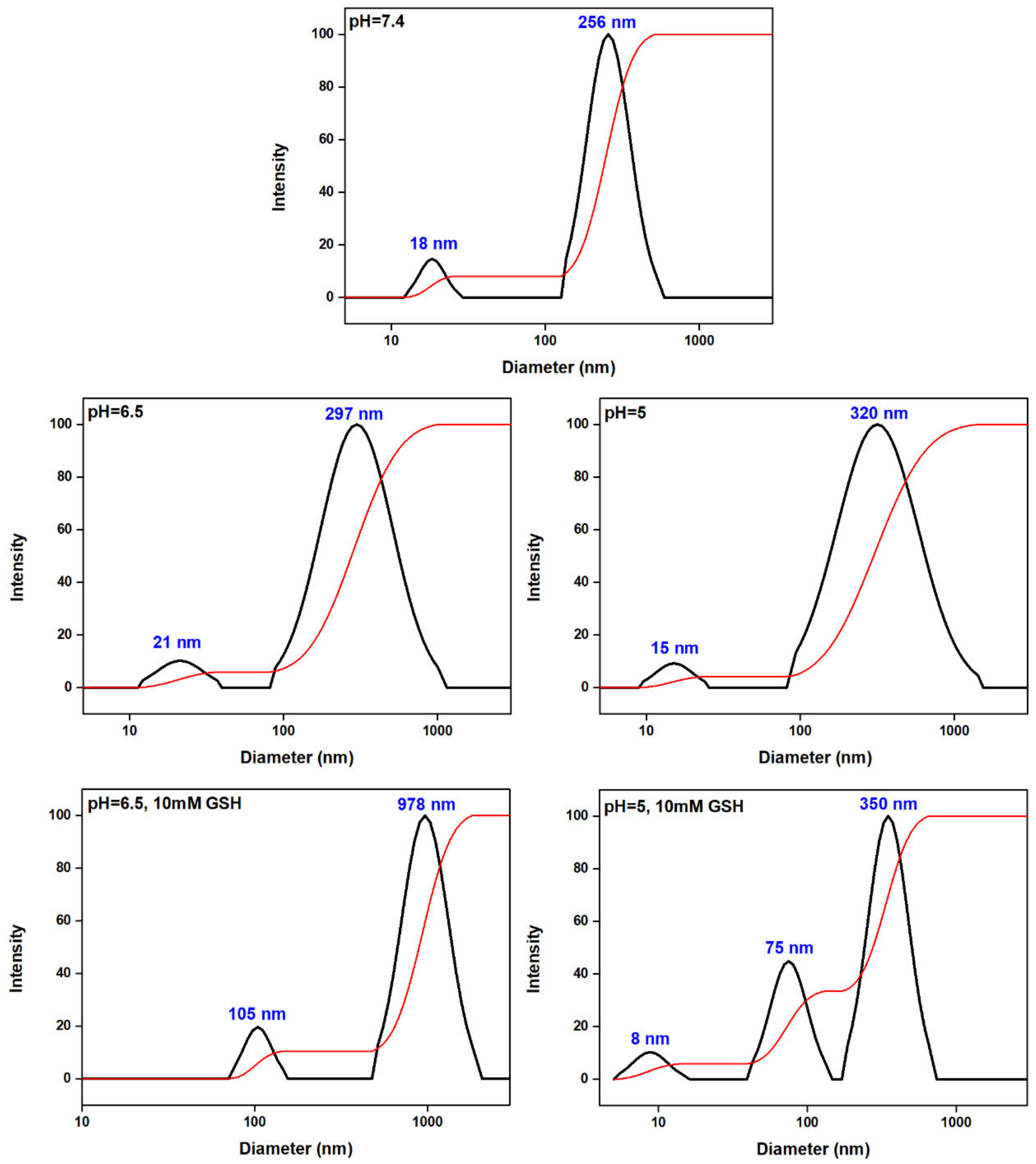

Figure S39. Schematic representation of the PCys10COPHis NPs size dependence from pH and GSH concentration, at 25 °C, at 90°.

DLS of the nanoparticles with encapsulated DOX

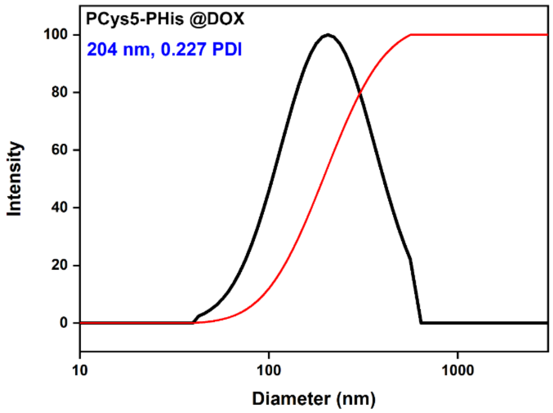

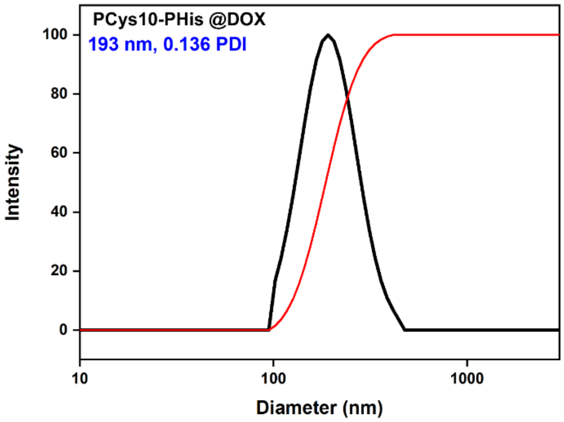

Figure S40. DLS measurements of the DOX loaded NPs of mPEO$_{227}$-*b*-P(Cys)$_x$-*b*-P(His)$_y$, at pH 7.4, 25 °C, 90°.

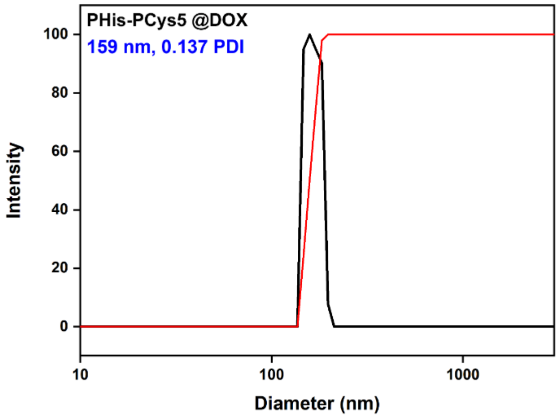

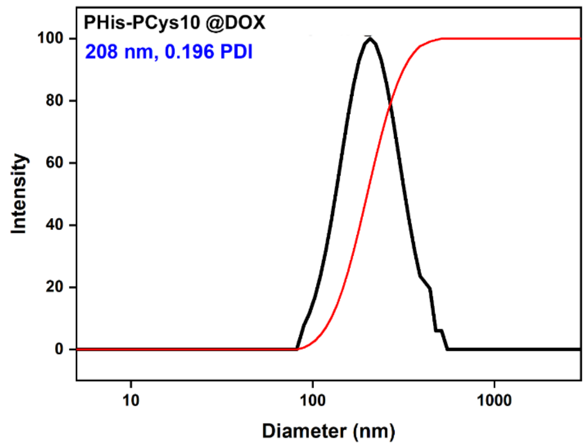

Figure S41. DLS measurements of the DOX loaded NPs of PHis-PCysX, at pH 7.4, 25 °C, 90°.

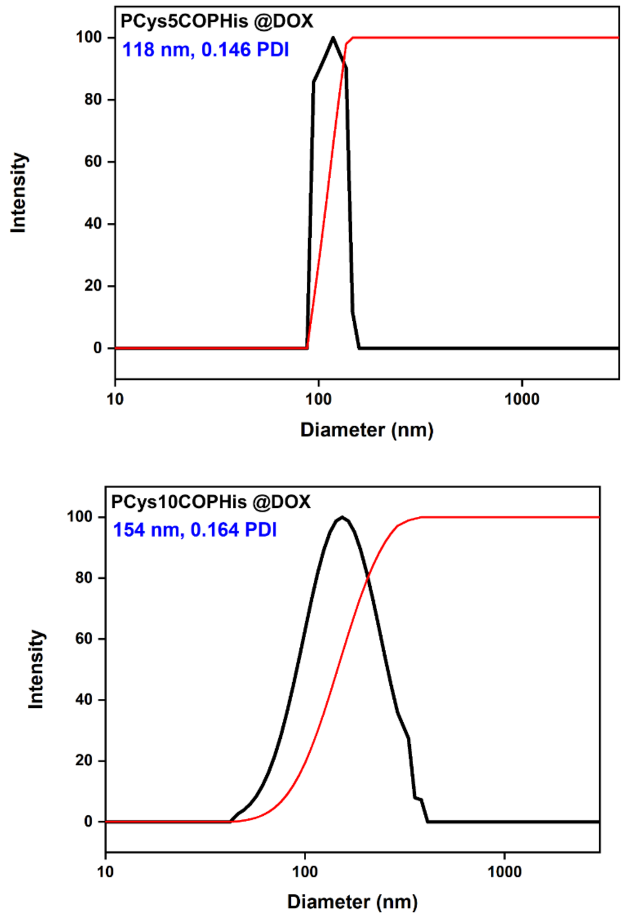

Figure S42. DLS measurements of the DOX loaded NPs of PCysXCOPHis, at pH 7.4, 25 °C, 90°.

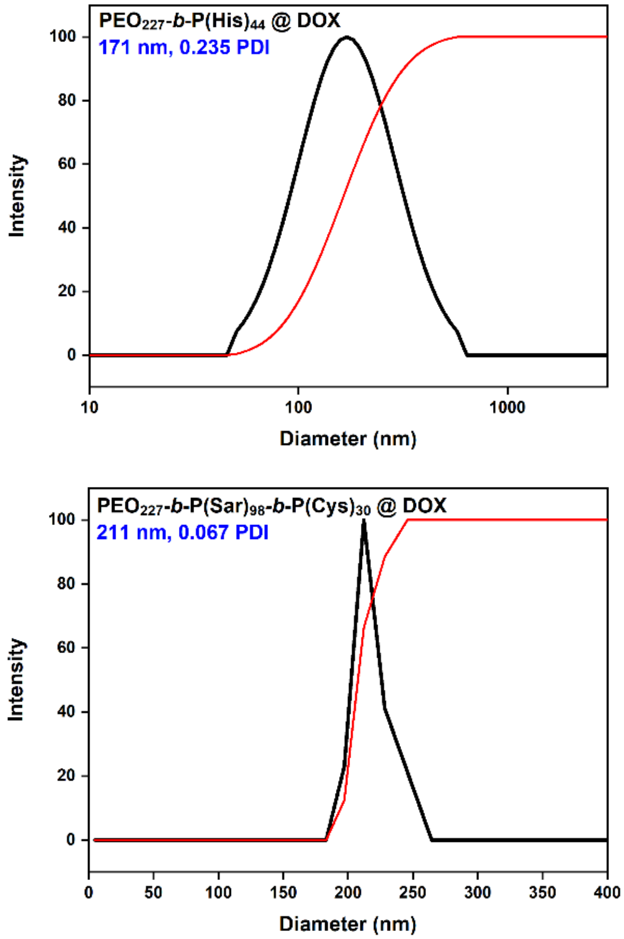

Figure S43. DLS measurements of the DOX loaded NPs of *m*PEO227-*b*-P(His)44 and *m*PEO227-*b*-P(Sar)98-*b*-P(Cys)30, at pH 7.4, 25 °C, 90°.

In vitro DOX release studies

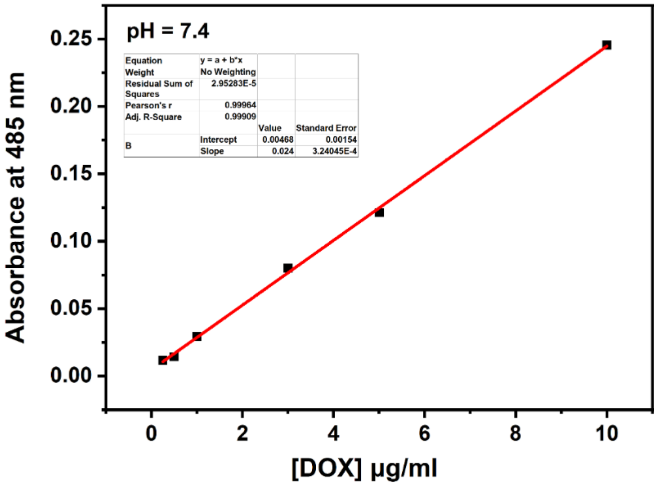

Figure S44. Calibration curve of doxorubicin at 485 nm in PBS buffer at pH = 7.4 with 150 mM NaCl, used for the calculation of EE% and LC% values.

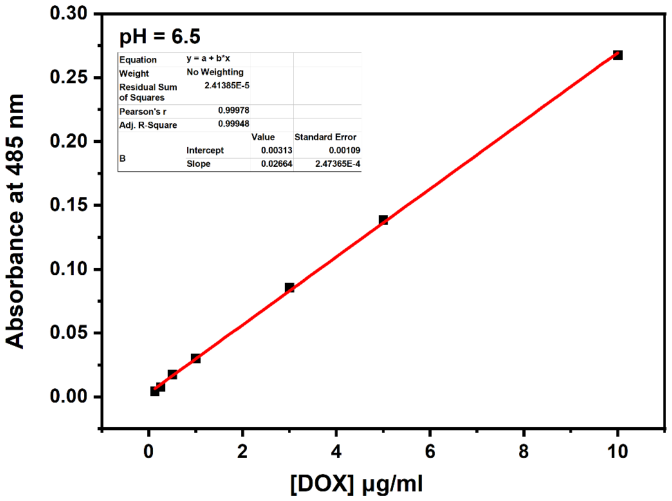

Figure S45. Calibration curve of doxorubicin at 485 nm in PBS buffer at pH = 6.5 with 150 mM NaCl.

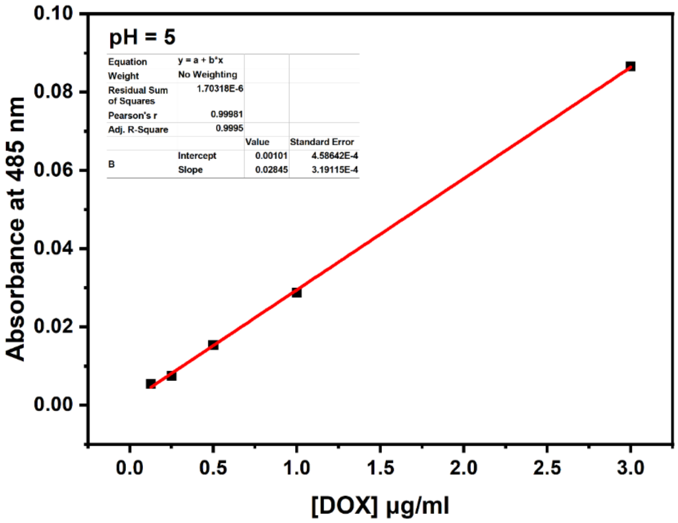

Figure S46. Calibration curve of doxorubicin at 485 nm in Acetate buffer at pH = 5 with 150 mM NaCl.

Release curves of the loaded nanoparticles.

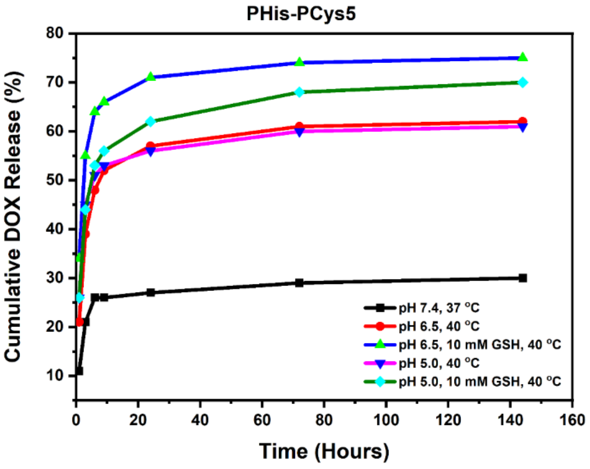

Figure S47. Cumulative Release of the DOX loaded PHis-PCys5 NPs.

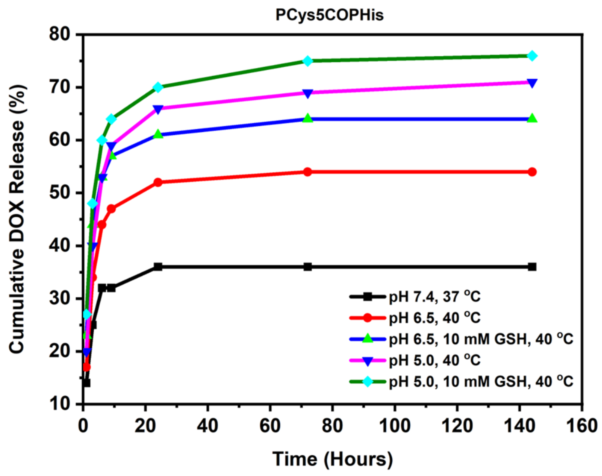

Figure S48. Cumulative Release of the DOX loaded PCys5COPHis NPs.

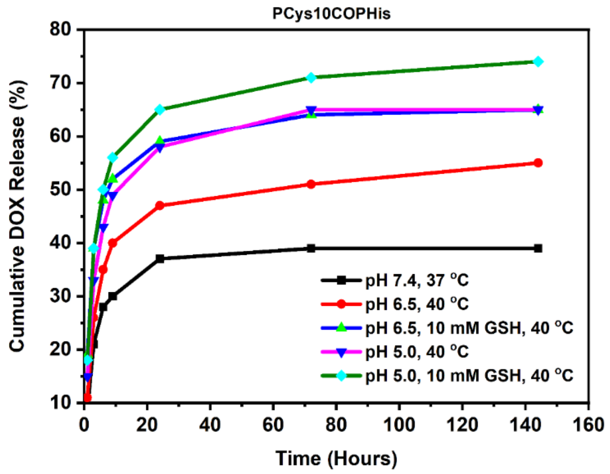

Figure S49. Cumulative Release of the DOX loaded PCys10COPHis NPs.

References

1. Mavrogiorgis, D.; Bilalis, P.; Karatzas, A.; Skoulas, D.; Fotinogiannopoulou, G.; Iatrou, H. Controlled polymerization of histidine and synthesis of well-defined stimuli responsive polymers. Elucidation of the structure–aggregation relationship of this highly multifunctional material. *Polymer Chemistry* **2014**, *5*, 6256-6278.
2. Habraken, G.J.; Koning, C.E.; Heuts, J.P.; Heise, A. Thiol chemistry on well-defined synthetic polypeptides. *Chemical communications* **2009**, 3612-3614.
3. Fetsch, C.; Grossmann, A.; Holz, L.; Nawroth, J.F.; Luxenhofer, R. Polypeptoids from N-Substituted Glycine N-Carboxyanhydrides: Hydrophilic, Hydrophobic, and Amphiphilic Polymers with Poisson Distribution. *Macromolecules* **2011**, *44*, 6746-6758, doi:10.1021/ma201015y.
4. Hadjichristidis, N.; Iatrou, H.; Pitsikalis, M.; Sakellariou, G. Synthesis of well-defined polypeptide-based materials via the ring-opening polymerization of α-amino acid N-carboxyanhydrides. *Chemical reviews* **2009**, *109*, 5528-5578.
5. Deming, T.J. Living polymerization of α-amino acid-N-carboxyanhydrides. *Journal of Polymer Science Part A: Polymer Chemistry* **2000**, *38*, 3011-3018.
6. Kricheldorf, H.R. Polypeptides and 100 years of chemistry of α-amino acid N-carboxyanhydrides. *Angewandte Chemie International Edition* **2006**, *45*, 5752-5784.
7. Aliferis, T.; Iatrou, H.; Hadjichristidis, N. Living polypeptides. *Biomacromolecules* **2004**, *5*, 1653-1656.
8. Pickel, D.L.; Politakos, N.; Avgeropoulos, A.; Messman, J.M. A mechanistic study of α-(amino acid)-N-carboxyanhydride polymerization: Comparing initiation and termination events in high-vacuum and traditional polymerization techniques. *Macromolecules* **2009**, *42*, 7781-7788.
9. Hadjichristidis, N.; Iatrou, H.; Pispas, S.; Pitsikalis, M. Anionic polymerization: high vacuum techniques. *Journal of Polymer Science Part A: Polymer Chemistry* **2000**, *38*, 3211-3234.

10. Uhrig, D.; Mays, J.W. Experimental techniques in high-vacuum anionic polymerization. *Journal of Polymer Science Part A: Polymer Chemistry* **2005**, *43*, 6179-6222.
11. Hadjichristidis, N.; Hirao, A. *Anionic Polymerization*; Springer: 2015.
